# EZH2 is overexpressed in transitional preplasmablasts and is involved in human plasma cell differentiation

**DOI:** 10.1038/s41375-019-0392-1

**Published:** 2019-02-12

**Authors:** Laurie Herviou, Michel Jourdan, Anne-Marie Martinez, Giacomo Cavalli, Jerome Moreaux

**Affiliations:** 1 IGH, CNRS, University of Montpellier, Montpellier, France; 2 Department of Biological Hematology, CHU de Montpellier, Montpellier, France; 3 UFR de Médecine, University of Montpellier, Montpellier, France

**Keywords:** Plasma cells, Differentiation

## Abstract

Plasma cells (PCs) play a major role in the defense of the host organism against pathogens. We have shown that PC generation can be modeled using multi-step culture systems that reproduce the sequential cell differentiation occurring in vivo. Using this unique model, we investigated the role of EZH2 during PC differentiation (PCD) using H3K27me3 and EZH2 ChIP-binding profiles. We then studied the effect of the inhibition of EZH2 enzymatic activity to understand how EZH2 regulates the key functions involved in PCD. EZH2 expression significantly increases in preplasmablasts with H3K27me3 mediated repression of genes involved in B cell and plasma cell identity. EZH2 was also found to be recruited to H3K27me3-free promoters of transcriptionally active genes known to regulate cell proliferation. Inhibition the catalytic activity of EZH2 resulted in B to PC transcriptional changes associated with PC maturation induction, as well as higher immunoglobulin secretion. Altogether, our data suggest that EZH2 is involved in the maintenance of preplasmablast transitory immature proliferative state that supports their amplification.

## Introduction

Plasma cells (PCs) are highly specialized cells representing the end stage of B cell differentiation. PCs play an important role in humoral immunity by synthesizing and secreting antibodies protecting the host against infections [1]. B to PC differentiation is a complex and highly coordinated process. The differentiation of B cells into PC is guided by the hierarchical expression of transcription factors (TFs) and is influenced by the microenvironment [2]. B cell master TFs including PAX5, BCL6 and BACH2 negatively regulate PC fate-determining TFs. Reciprocally, IRF4, BLIMP1 and XBP1 PC TFs are required to suppress the B cell lineage genes, as well as to activate the antibody-secreting cell (ASC) program [2, 3]. Although the role of this complex network of TFs has been investigated, the mechanisms regulating key transcriptional steps in PC differentiation remain poorly known. We have developed a multi-step culture system, modeling B to PC differentiation, where various combinations of cytokines and activation molecules are used to reproduce the sequential PC differentiation occurring in the different organs/tissues in vivo. In this culture model, memory B cells (MBCs) differentiate into CD20^low/−^ CD38^−^ preplasmablasts (prePBs), CD20^−^CD38^+^CD138^−^ plasmablasts (PBs), CD20^−^CD38^+^CD138^+^ early PCs and, finally, into long-lived PCs (LLPCs), which may survive and produce continuously high amounts of immunoglobulins (Igs) 2 months in vitro [4–6]. PrePBs have been identified in lymph nodes, tonsil and bone marrow in human [6, 7]. This transitional stage is characterized by the absence of CD20, CD38, and CD138 markers and the coexpression of B and PC TFs, but at a reduced level compared with B cells, PBs, or PC [6]. The phenotype of in vitro*-*generated PBs and early PCs is similar to the phenotype of the PBs detected in the peripheral blood [4, 6]. It is thought that cellular transitions during development are mostly driven by epigenetic and transcriptional changes of a selective group of genes. However, the terminal differentiation of B lymphocytes into PC is a unique process whose epigenetic modifications remain poorly understood.

DNA methylation has been shown to be largely remodeled during PC differentiation (PCD). Indeed, B cell engagement toward PC phenotype is associated with cell division-dependent heterochromatin DNA-demethylation, hypermethylation of Polycomb-rich regions and 5-hydroxymethylation of enhancers and genes involved in PCD, such as BLIMP1 [8, 9]. We recently showed that several miRNAs could also participate into the regulation of expression of key transcription factors during PCD, including IRF4, PRDM1, ELL2, and ARID3A [10]. Moreover, B cell and PC transcription factors can cooperate with epigenetic enzymes, such as histone deacetylases or methyltransferases, to regulate their target genes [11, 12]. Enhancer of Zeste Homolog 2 (EZH2), the catalytic subunit of Polycomb Repressive Complex 2 (PRC2), is able to trimethylate the lysine 27 in histone H3 (H3K27me3) to repress transcription. EZH2 has been shown to play an important role in germinal center formation in mice. Indeed, EZH2 is overexpressed in germinal center B cells, and induces their proliferation through repression of cell cycle inhibitors, such as CDKN1A and CDKN1B [13–15]. It has been suggested that EZH2 protects activated B cells from AID-dependent DNA damage induced apoptosis [16]. Moreover, in these cells, EZH2 transiently represses B cell differentiation by inhibiting key PC genes such as IRF4 and BLIMP1 [13]. EZH2 can also acts as a partner of BCL6 in germinal center cells to repress its target genes [16]. In mice, B to PC differentiation is associated with transcriptional and epigenetic regulation related to cell division-coupled chromatin accessibility changes [17]. In this study, we aimed to define EZH2 target genes and thus understand its role in normal human PC differentiation, using our previously described in vitro model [4–6]. Our data indicate that EZH2 is overexpressed in the transitional prePBs stage, where it represses both B cell and the PC transcriptional programs. EZH2 inhibition using specific inhibitor EPZ-6438 induces an early derepression of mature PC gene signature, leading to an accelerated differentiation of MBC into competent antibody-secreting cells. We propose a model in which EZH2 is involved in the maintenance of prePBs transitory immature proliferative state to support their amplification prior PC differentiation.

## Materials and methods

### Reagents

Human recombinant interleukin (IL)-2 was purchased from R&D Systems (Minneapolis, MN, USA), interferon-alpha-2b (IFN-α, IntronA) from Merck Canada Inc. (Kirckland, Canada), IL-6, IL-10, and IL-15 from PeproTech (Rocky Hill, NJ, USA). Used antibodies are listed in Supplementary Table 1.

### Cell samples

Peripheral blood cells from healthy volunteers were purchased from the French Blood Center (Toulouse, France) and CD19+ CD27+ MBCs were purified (≥95% purity) as described [4].

### Cell cultures

MBCs were differentiated using a previously described culture protocol [4, 6, 18]. All cultures were performed in Iscove modified Dulbecco medium (Invitrogen, Carlsbad, CA, USA), 10% FCS and 25–35% of Resto-6 cells supernatant. 1.5 × 10^5^/ml purified peripheral blood MBCs were activated for 4 days by 10 µg/ml of phosphorothioate CpG oligodeoxynucleotides (ODN) 2006 (Sigma-Aldrich, St Louis, MO, USA), 50 ng/ml histidine tagged soluble CD40 ligand (CD40L) and 5 µg/ml of an anti-poly-histidine mAb (R&D Systems) in the presence of 20 U/ml IL-2, 50 ng/ml IL-10, and 10 ng/ml IL-15. The next 3 days, PBs were generated by removing ODN and CD40L and changing the cytokine cocktail (20 U/ml IL-2, 50 ng/ml IL-6, 50 ng/ml IL-10, and 10 ng/ml IL-15). From day 7 to day 10, PBs were differentiated into early PCs by adding 50 ng/ml IL-6, 10 ng/ml IL-15, 500 U/ml IFN-α. EPZ-6438 (1 µM) (EPIZYME, Cambridge, MA, USA), GSK-126 (2 µM) (GlaxoSmithKilne, Brentford, UK) or MAK-683 (2 µM) (Novartis Pharmaceuticals, Basel, Switzerland) were added at the start of each step and their effects were evaluated by analyzing cell counts and phenotype at the end of each step. mRNA expression data are available at ArrayExpress (http://www.ebi.ac.uk/arrayexpress/, E-MTAB-1771, E-MEXP-2360 and E-MEXP-3034) [4, 6].

### Cell viability

Cell concentration and viability were assessed using the trypan blue dye exclusion assay.

### Cell cycle analysis

Cycling cells were assessed using DAPI staining (Sigma-Aldrich). S-phase cells were visualized by bromodeoxyuridine (BrdU) incorporation. BrdU incubation was carried out for 1 h before fixation and labeling with an anti-BrdU antibody (APC BrdU flow kit, BD Biosciences, San Jose, CA, USA) according to the manufacturer’s instructions.

### Study of apoptosis

After incubation with EPZ-6438 (1 uM) or DMSO, cells were washed twice in PBS and apoptosis was assayed with PE-conjugated Annexin V labeling (BD Pharmigen). Fluorescence was analyzed on a LSR Fortessa X20 flow cytometer (Becton Dickinson).

## Results

### EZH2 is significantly upregulated in preplasmablasts during PCD

**Fig. 1:**
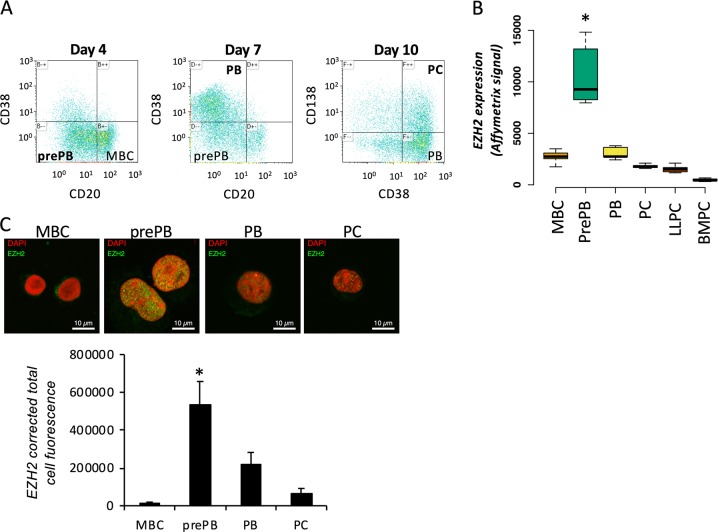
*EZH2* is overexpressed in preplasmablasts: **a** PCD in vitro model highlighting CD20, CD38, and CD138 expression in memory B cells (MBC), pre-plasmablasts (prePB), plasmablasts (PB) and plasma cells (PC). **b** *EZH2* Affymetrix microarrays expression signal during PCD, in MBCs, prePBs, PBs, PCs, long-lived plasma cells (LLPC) and bone marrow plasma cells (BMPC). **c** EZH2 protein levels, assessed by immunofluorescence, in MBCs (Day 0), prePBs (Day 4), PBs (Day 7) and PCs (Day 10), using an anti-EZH2 antibody. Corrected total cell fluorescence (CTCF) was assessed using the ImageJ software (mean number of cells counted: 40)

Affymetrix microarrays were used to assess EZH2 expression in our in vitro model of normal PCD (Fig. 1a). EZH2 expression is significantly increased in the prePB stage of the PCD (*p* < 0.001) (Fig. 1b). Interestingly, other members of PRC2 core complex, like EED or SUZ12, follow the same expression pattern as EZH2 (*p* < 0.001) (Supplementary Fig. S1). EZH2 expression at the protein level is 40, 2, and 8 times more elevated in prePBs compared to MBCs, PBs, and PCs, respectively (*p* < 0.001) and positively correlated with *EZH2* mRNA levels in each population (Fig. 1b, c). Surprisingly, H3K27me3 global levels did not correlate with EZH2 expression levels and were stable during PCD (Supplementary Fig. S2). However, the histone methyltransferase EZH1 can also catalyze H3K27me3. Interestingly, EZH1 and EZH2 expression levels were anti-correlated from MBC to PC stage (*p* = 0.0035) (Supplementary Fig. S3). This EZH1 prePB-specific downregulation might partly explain the relative stability of H3K27me3 levels during PCD while EZH2 is upregulated at this stage.

### EZH2 regulates B cell gene signatures during human PCD

Since a core PRC2 member displays an increased expression in prePBs and in PBs, EZH2 and H3K27me3 chromatin immunoprecipitations followed by sequencing (ChIP-Seq) were performed in order to identify their target genes in these cell populations. A genome distribution analysis of EZH2 and its H3K27me3 deposited mark confirmed previously published results showing an enrichment at promoters, intronic and distal intergenic regions (Supplementary Fig. S4 and Supplementary Table S2). The specific enrichment of H3K27me3 and EZH2 around transcription start sites (TSSs) (Supplementary Fig. S5) also confirmed the known function of PRC2 as a major transcriptional regulator [19]. Interestingly, Gene Ontology analysis of H3K27me3-marked genes revealed a significant enrichment of genes involved in developmental processes (Supplementary Fig. S6 and Supplementary Table S3). As expected, EZH2 and H3K27me3 are recruited on genes involved in embryonic development, such as the *HOX* gene clusters, and genes regulating neurogenesis or development of other tissues (Supplementary Fig. S6). These results therefore confirmed the role of PRC2 in repressing genes involved in developmental processes during cell differentiation [20].

**Fig. 2:**
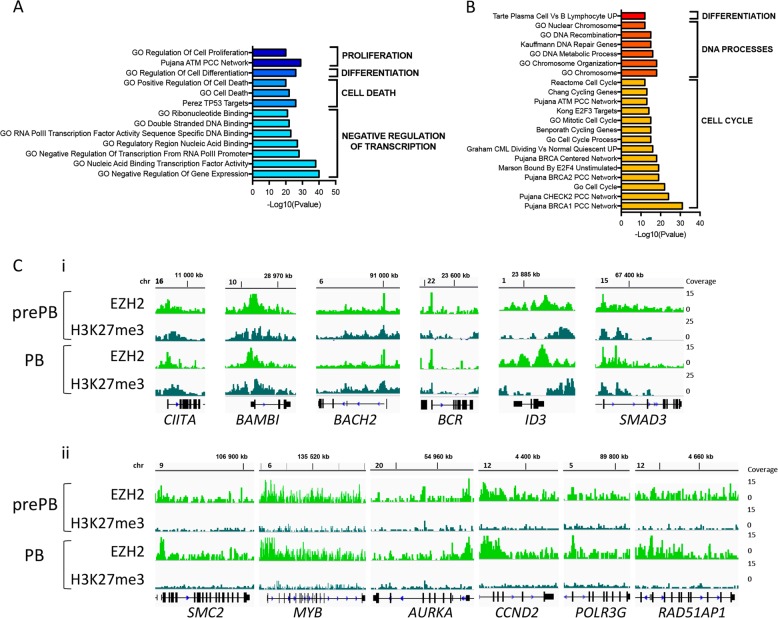
EZH2 regulates memory B cell gene signature during PCD: **a** GSEA enriched pathways of genes upregulated in MBC and associated with H3K27me3 in prePB and/or PB. Log10(pvalue) was assessed for each pathway (FDR ≤ 0.05). **b** GSEA enriched pathways of genes downregulated in MBC and associated with EZH2o in prePB and/or PB. Log10(pvalue) was assessed for each pathway (FDR ≤ 0.05). **c** i: IGV visualization of EZH2 and H3K27me3 enrichment on *CIITA*, *BAMBI, BACH2*, *BCR*, *ID3*, and *SMAD3* genes in prePBs and PBs. ii: Genomic snapshots of EZH2 and H3K27me3 ChIP-seq results on *SMC2*, *MYB, AURKA*, *CCND2*, *POLR3G*, and *RAD51AP1* genes in prePBs and PBs

Gene expression analysis showed that 30.6% of MBC specific genes were associated with EZH2-associated H3K27me3 in prePBs and PBs. Moreover, these genes were significantly downregulated in prePBs and PBs compared with MBC (Supplementary Fig. S8A and Supplementary Table S4). These results suggest that, upon MBC activation, EZH2 represses these genes in prePBs and PBs through H3K27me3 deposition. GSEA pathway analysis demonstrated a significant enrichment of genes involved in negative regulation of proliferation, differentiation and cell death, as well as in negative regulation of transcription (Fig. 2a and Supplementary Table S4). Notably, H3K27me3-associated repressed genes in prePB/PB were found to be key known B-cell fate genes including *CIITA*, *BAMBI*, *BACH2*, *BCR*, *ID3*, or *SMAD3* (Fig. 2c).

Surprisingly, 21.5% of EZH2-bound promoters were not enriched with H3K27me3 repressive histone mark (EZH2o) (Fig. 2c and Supplementary Table S2). EZH2o-associated gene promoters were enriched in DNA-binding motifs for transcription factors involved in different processes, notably B cell differentiation, including NFAT, SP1, MYC, c-MYB, SMAD, or C/EBP (Supplementary Fig. S7 and Supplementary Table S6). Interestingly, *CREB1, E2F1, GABPβ2, c-MYB, NFATC3, NRF1*, and *YY1* expression positively correlated with *EZH2* expression from MBC to BMPC, whereas *ETS2* and *RORA* expression where anti-correlated to EZH2 levels. HIF1α and EZH2 expression significantly anti-correlated from MBC to early PC (Supplementary Fig. S7). These data suggest that EZH2 and these transcription factors could potentially regulate a common set of genes involved in PCD.

Notably, 13.8% of prePB/PB-up-regulated genes compared with MBC were associated with EZH2o in these cells. These genes were found to be repressed in MBCs and significantly upregulated at early stages of PCD (Supplementary Fig. S8B and Supplementary Table S4). Pathway analysis highlighted a significant enrichment of genes upregulated in PC compared with B cells, involved in DNA processes such as DNA repair or chromosome organization, and in cell cycle (Fig. 2b, c and Supplementary Table S5). These results suggest that EZH2 could directly repress a B cell transcriptional program in prePB/PB during cell activation and proliferation induction.

### EZH2 is involved in the regulation of the PC transcriptional program

**Fig. 3:**
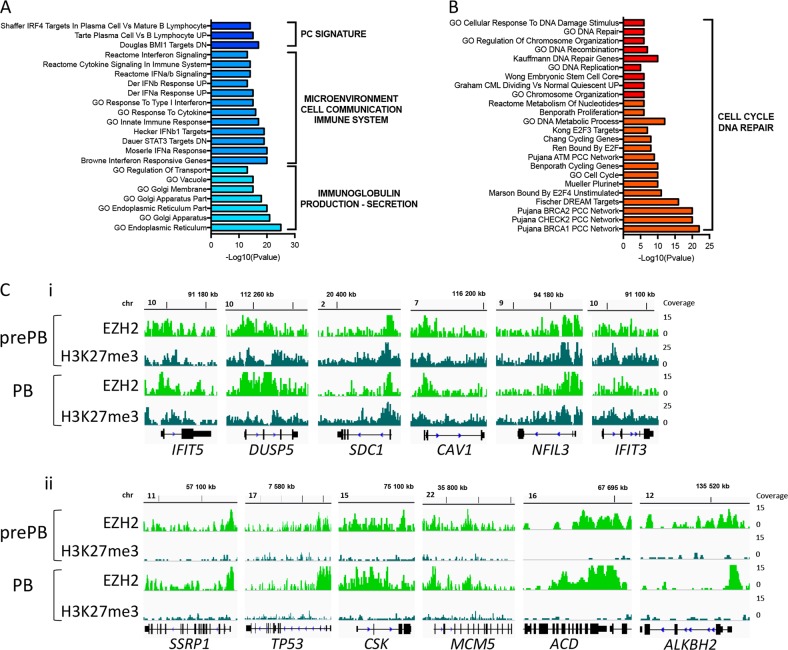
EZH2 regulates plasma cell transcriptional program during PCD: **a** GSEA enriched pathways of genes upregulated in PC and associated with H3K27me3 in prePBs and/or PBs. Log10(pvalue) was assessed for each pathway (FDR ≤ 0.05). **b** GSEA enriched pathways of genes downregulated in PC and associated with EZH2o in prePBs and/or PBs. Log10 (*p* value) was assessed for each pathway. **c** i: Genomic snapshots of EZH2 and H3K27me3 enrichment on *IFIT5*, *DUSP5, SDC1*, *CAV1*, *NFIL3*, and *IFIT3* genes in prePBs and PBs. ii: Genomic snapshots of EZH2 and H3K27me3 ChIP-seq results on *SSRP1*, *TP53, CSK*, *MCM5*, *ACD*, and *ALKBH2* genes in prePBs and PBs

30.5% of PC signature genes were associated with H3K27me3 in prePB/PB. These genes were found to be significantly upregulated in PC compared with prePB/PB (Supplementary Fig. S9A and Supplementary Table S4), suggesting that EZH2 could participate in PC transcriptional program repression during the more immature stages of the PCD. Pathway analysis revealed that these H3K27me3-associated genes were enriched in key PC IRF4 transcription factor targets (*DUSP5, CAV1, NFIL3, UAP1, PAM, CFLAR, FKBP11, UBE2J1, TIMP2, GFPT1, SLAMF7, BMP6, AVPI1, CFLAR, and BSPRY*) [21], cell to cell communication including *CD138/SDC1* and immune response (*IFIT5*, *IFIT3*). Moreover, some of these genes were involved in endoplasmic reticulum and Golgi apparatus functions that play a major role in PC to accommodate the synthesis of secreted Ig (Fig. 3a, c and Supplementary Table S5). According to these data, EZH2 through H3K27me3 appears to participate in key PC transcriptional program regulation including IRF4 targets, CD138/SDC1 and Ig secretion stress adaptation.

EZH2o-associated gene represented 18% of the genes that were significantly upregulated in prePB/PB and repressed in PC (Supplementary Fig. S9B and Supplementary Table S4). These genes were enriched in genes involved in cell cycle, including *CSK* and *MCM5* genes, and in DNA damage repair processes, such as *SSRP1*, *TP53*, *ACD*, and *ALKBH2* genes (Fig. 3b, c and Supplementary Table S5). These results suggest an EZH2 role in repressing key features of PC in prePBs, thus poising differentiation, while associating with active characteristic prePB/PB genes promoting cell proliferation.

### EZH2 targeting affects B to plasma cell gene expression profiles

**Fig. 4:**
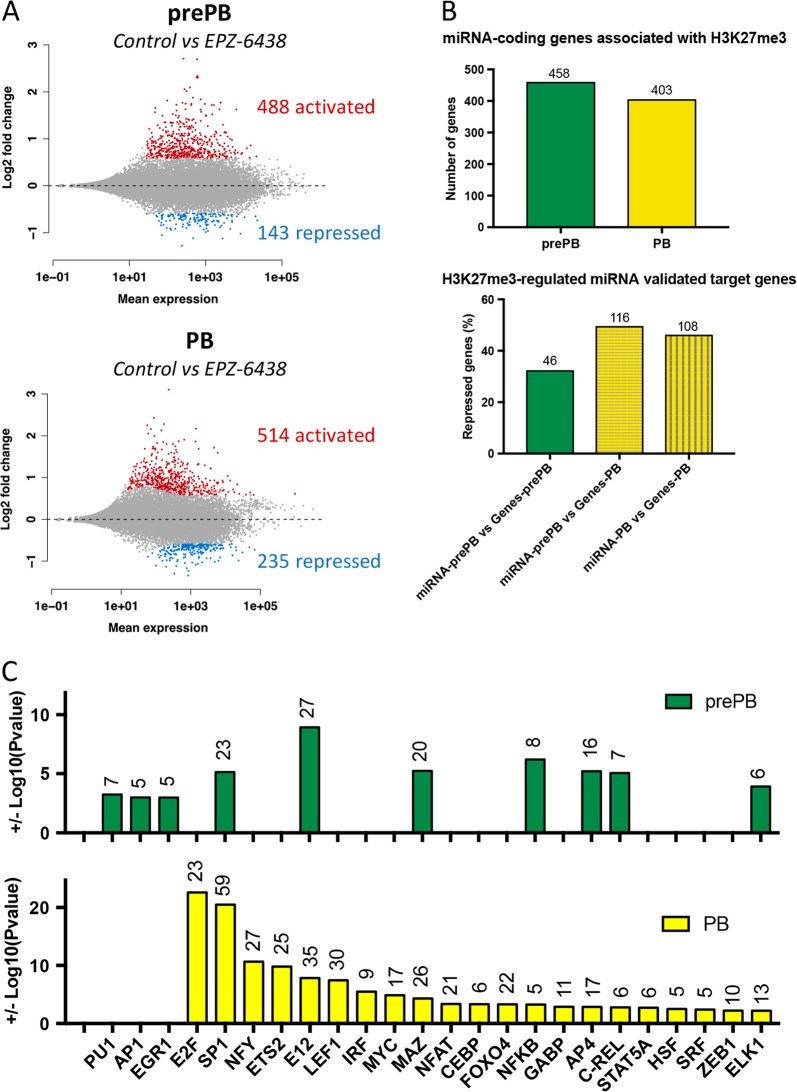
EPZ-6438 treatment affects gene expression during PCD: **a** Scatterplots of EPZ-6438-deregulated genes in prePBs and PBs. Activated genes are represented in red, while repressed genes are represented in blue. **b** First bar plot represents the number of miRNA-coding genes associated with H3K27me3 in prePBs and/or PBs. Second bar plot represents the percentage (*Y*-axix) and number (top of each bar) of EPZ-6438-activated genes that are validated targets of H3K27me3-associated miRNAs. H3K27me3-associated miRNA validated targets in prePBs were compared with EPZ-6438-activated genes in prePBs (green bar) and PB (yellow-horizontal stripes bar); and H3K27me3-associated miRNA validated targets in PB were compared with EPZ-6438-activated genes in PBs (yellow-vertical stripes bar). **c** Transcription factors predicted to recognize and regulate EPZ-6438-repressed genes in prePBs and/or PBs. Log10 (*p* value) was assessed for each transcription factors

To better understand the role of EZH2 in PCD, the chemical inhibitor EPZ-6438 was used to inhibit its catalytic activity. The drug was added at each step of the in vitro system, as illustrated by the chart in Supplementary Fig. S10, to avoid any reversibility of the significant drug-induced global H3K27me3 decrease (Supplementary Figs. S11 and S12). RNA sequencing of prePBs, PBs and PCs treated or not with EPZ-6438 was performed. In prePBs, 488 genes were significantly activated after treatment, while 143 were repressed (ratio ≥ 1.5, FDR ≤ 0.05; Fig. 4a and Supplementary Table S7). In PBs, 514 genes were upregulated whereas 235 where downregulated after treatment (ratio ≥ 1.5, FDR ≤ 0.05; Fig. 4a and Supplementary Table S7). Moreover, only 183 genes (over 488 in prePB and 514 in PB) were commonly activated in prePBs and PBs, while 24 genes were similarly downregulated in both cell types (Supplementary Figs. S13 and S14). This high proportion of genes specifically deregulated in one cell stage suggest that EZH2 could regulate unique transcriptional programs in prePBs and in PBs, respectively. Interestingly, EPZ-6438 treatment had a more modest effect on the PC transcriptional profile: 42 genes were found upregulated while 12 were repressed (ratio ≥ 1.5, FDR ≤ 0.05; Supplementary Fig. S15 and Supplementary Table S7). Altogether, these data underscore that EZH2 main functions involve the prePB and PB cell stages. Almost all EPZ-6438-activated genes were associated with H3K27me3 in prePBs (84%) and PBs (79%), implying that EZH2 directly represses their expression in immature stages of the PCD (Fig. 4b). EPZ-6438-induced gene repression might involve a direct or indirect mechanism. Less than 10% of EPZ-6438-downregulated genes were associated with EZH2 without H3K27me3 (Supplementary Table S8). This low percentage suggests that EZH2 regulates expression of those genes mostly indirectly. EZH2-mediated H3K27me3 has been previously shown to repress miRNA expression. Our results show that 458 and 403 miRNAs where enriched in H3K27me3 repressive mark in prePBs and PBs, respectively (Fig. 4b and Supplementary Table S9). Using a previously described R package miRTarget [10], we identified H3K27me3-associated miRNAs validated targets (Supplementary Table S9). Interestingly, 32.2% of EPZ-6438-downregulated genes in prePBs are potential repression targets of H3K27me3-associated miRNAs in prePBs. Similarly, 49.6% of EPZ-6438-downregulated genes in PB might be repressed by H3K27me3-associated miRNAs in prePB, and 46% by H3K27me3-associated miRNAs in PBs (Fig. 4b and Supplementary Table S9). Moreover, several of these miRNAs were found to potentially target important B cell transcription factors repressed under EPZ-6438 treatment: PAX5 (miR-1270, miR-4710, miR-3714, miR-4739), MYB (miR-198, miR-429), and CIITA (miR-4257, miR-4270, miR-4739, miR-650) (Supplementary Table S9). Interestingly, none of these miRNAs were previously described as playing a role in PCD [10]. EZH2 could also regulate transcription factor expression, which could in turn modulate gene transcription. Binding motif analysis showed that EPZ-6438-repressed genes could be targeted by different transcription factors involved in PCD, including the E2F family, IRF family, MYC, NF-κB, or STAT5A (Fig. 4c and Supplementary Table S10). Interestingly, the expression of the transcription factors *RELB* in prePBs and *E2F1*, *E2F7*, and *IRF5* in PBs was found to be downregulated, and the expression of the transcription factors *IRF1* in prePB and *CEBPD* in PB was upregulated after EPZ-6438 treatment (Supplementary Table S7). Deregulation of these transcription factors could thus take part in the downregulation of their potential target genes after EPZ-6438 treatment.

**Fig. 5:**
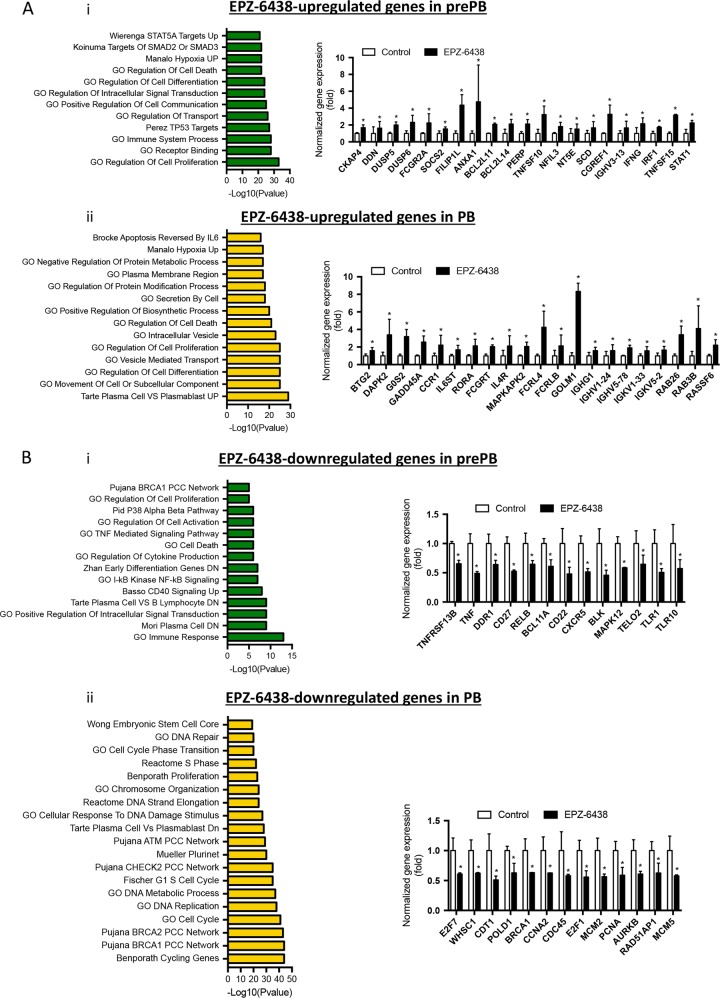
Function of EPZ-6438 target genes in preplasmablasts and plasmablasts: **a** i: First chart represents Log10(pvalue) of GSEA enriched pathways of EPZ-6438-upregulated genes in prePBs (FDR ≤ 0.05). Second chart represent the fold expression (EZP-6438 condition over control) of genes related to first chart pathways. ii: First chart represents Log10(pvalue) of GSEA enriched pathways of EPZ-6438-upregulated genes in PBs (FDR ≤ 0.05). Second chart represent the fold expression (EZP-6438 condition over control) of genes related to first chart pathways. **b** i: First chart represents Log10 (*p* value) of GSEA enriched pathways of EPZ-6438-downregulated genes in prePBs (FDR ≤ 0.05). Second chart represent the fold expression (EZP-6438 condition over control) of genes related to first chart pathways. ii: First chart represents Log10 (*p* value) of GSEA enriched pathways of EPZ-6438-downregulated genes in PBs (FDR ≤ 0.05). Second chart represent the fold expression (EZP-6438 condition over control) of genes related to first chart pathways. Statistical significance between conditions was assessed using Student paired *t*-test (*p value < 0.05)

To further understand the role of EZH2 in prePBs and PBs, we performed GSEA pathway enrichment analysis. EPZ-6438-upregulated genes in prePBs were enriched in STAT5A (such as *CKAP4*, *DDN*, *DUSP5*, *FCGR2A*, or *SOCS2*), SMAD2/3 (including *NFIL3*, *NT5E*, and *SCD*) and TP53 targets, in genes involved in hypoxia (including *DUSP6* and *FILIP1L*), cell death (such as *ANXA1*, *BCL2L11*, BCL2L14, PERP, or TNFSF10), cell differentiation (for examples IRF1 and STAT1), communication and proliferation (such as *CGREF1*), and immune response (including *IGHV3-13*, *IFNG*, and *TNFSF15*) (Fig. 5a i and Supplementary Table S11). Genes upregulated in PBs after EPZ-6438 treatment were mostly involved in hypoxia, protein metabolism, cell death (such as *BTG2*, *DAPK2*, *G0S2*, *GADD45A*, or *RASSF6*), cell differentiation (such as *CCR1*, *IL6ST* or *RORA*) vesicle transport and cell secretion (including *FCGRT*, *GOLM1*, *RAB26*, and RAB3B) (Fig. 5 ii). Moreover, these genes were more highly expressed in mature PC compared with PBs (such as *IL4R* or *MAPKAPK2*). Immunoglobin genes (including *IGHG1*, *IGHV1-24*, *IGHV5-78*, *IGKV1-33*, or *IGKV5-2*) were also found to be significantly upregulated after treatment (Fig. 5a and Supplementary Table S11). EPZ-6438-repressed genes in prePBs are involved in cell proliferation (such as *DDR1* or *TELO2*) and pathways involved in B cell activation such as p38α/β pathway (including *BLK* and *MAPK12*), TNF signaling pathway (such as *TNFRSF13B* or *TNF*), NF-κB signaling (such as *CD27* or *RELB*), CD40 signaling, and cytokine production (such as *TLR10*). Moreover, specific B cell genes were identified (including *BCL11A*, *CD22*, *CXCR5*, and *TLR1*) (Fig. 5bi and Supplementary Table S10). EPZ-6438-repressed genes in PBs were involved in cell cycle regulation (such as *E2F7*, *CCNA2*, *E2F1*, or *AURKB*), DNA replication (including *CDT1*, *POLD1*, *CDC45*, *MCM2*, and *MCM5*) and DNA damage response (such as *WHSC1*, *BRCA1*, *PCNA*, *RAD51AP1*). They were found to be also downregulated in mature PCs compared with PBs (Fig. 5bii and Supplementary Table S10). However, EPZ-6438-repressed genes in PBs that were involved in MBC maintenance were not significantly enriched in EZH2 or H3K27me3, suggesting that their repression might result from an indirect effect, possibly depending on derepression of PC specific genes that are known to repress MBC fate [1–4].

**Fig. 6:**
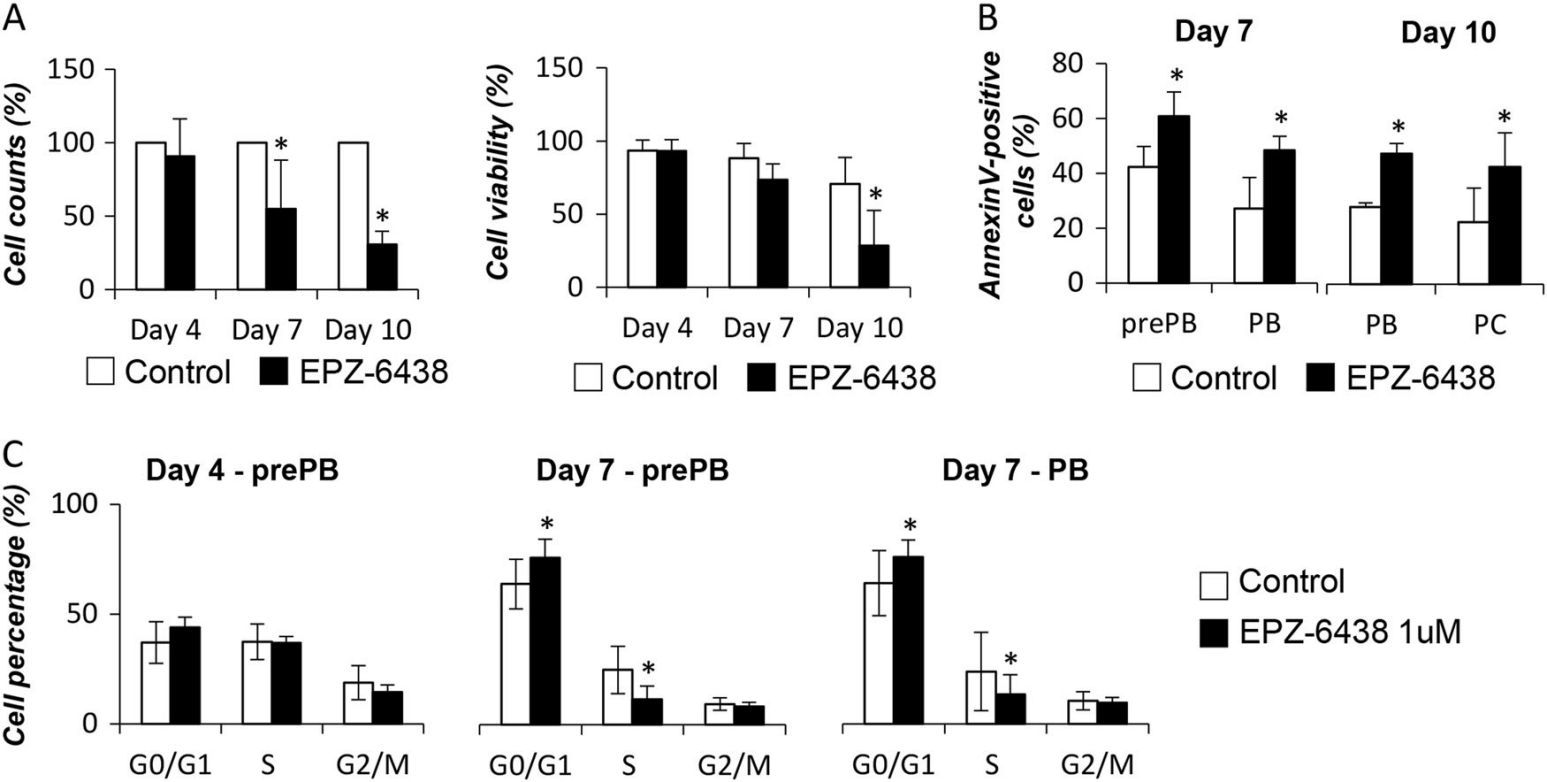
EPZ-6438 alters preplasmablasts and plasmablats viability and proliferation: **a** After EPZ-6438 treatment (1 uM), cell counts and viability were analyzed by trypan blue assay. Represented data are the mean percentage of the absolute counts or viability ± SD of 7 independent experiments. **b** Apoptosis induction was analyzed with AnnexinV-PE staining by flow cytometry. Represented data are the mean percentage values ± SD of 4 (Day 7) and 3 (Day 10) separate experiments. **c** Cell cycle was analyzed by flow cytometry using DAPI, BrdU incorporation and labeling with an anti-BrdU antibody. Represented data are the mean percentage values ± SD of 4 independent experiments. Statistical significance between conditions was assessed using Student paired *t*-test (*p value < 0.05)

At a cellular level, EPZ-6438 treatment had no effect on Day 4 cell counts nor cell viability (data not shown). At Day 7 and 10, global cell counts significantly decreased by 46% and 70%, respectively (Fig. 6a). According to these data, cell viability dropped to 73% and 29% (Fig. 6a). EZH2 inhibition induced a significantly increased apoptosis in prePBs (18.5%) and PBs (21,3%) at Day 7, and in PBs (36,3%) and PCs (37,6%) at Day 10 (Fig. 6b). Investigating caspase 3/7 activation, no significant difference was noted at day 7 whereas EZH2 inhibition results in capsase 3/7 activation at day 10 (Supplementary Fig. S23). Furthermore, EPZ-6438 treatment induced a cell cycle arrest of prePBs and PBs at Day 7 with a significant reduction of BrdU incorporation and an accumulation in the G_0_G_1_ cell cycle phase (*P* < 0.05) (Fig. 6c). The effect on proliferation was confirmed using CFSE assay, showing a significant decrease of the number of cell divisions in prePBs and PBs at Day 7 (Supplementary Fig. S16). Interestingly, a decreased number of 53BP1 foci per cell in PBs and PCs was observed at Day 7 and 10, respectively (Supplementary Fig. S17). This data could be the result of the observed cell cycle blockage and thus of a decreased replicative stress. Altogether, these results demonstrate a major role of EZH2 in transcriptionally regulating prePBs and PBs proliferation and survival.

### EZH2 inhibition affects plasma cell differentiation through stimulation of maturation

**Fig. 7:**
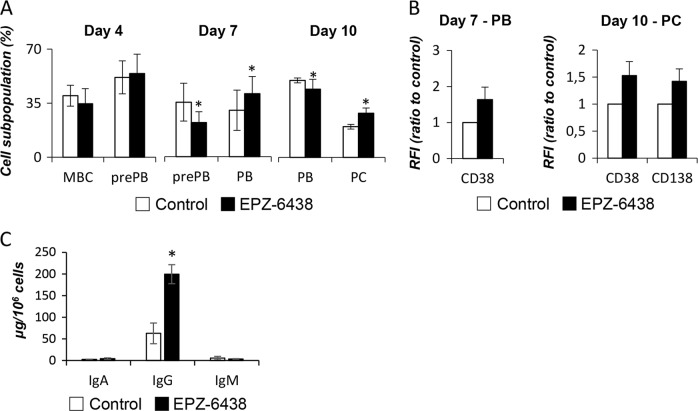
EZH2 inhibition accelerates PCD through gene expression regulation: **a** Mean percentage ± SD (7 separated experiments) of MBC and prePB at Day 4, prePBs and PBs at Day 7; and PBs and PCs at Day 10 after EPZ-6438 (1 μM) treatment. **b** Protein expression of surface markers CD38 in PBs (Day 7) and CD38 and CD138 in PCs (Day 10) was assessed by flow cytometry with or without EPZ-6438 (1 μM) treatment. Results are mean values of the relative fluorescence intensity (RFI) ± SD of viable cells of 7 independent experiments. **c** IgM, IgA, and IgG secretions by CD138 + PCs were assessed by ELISA and results are the mean immunoglobulin production in micrograms per day and per 10^6^cells determined in 3 separate experiments. Statistical significance between conditions was assessed using Student paired *t*-test (*p value < 0.05)

Hierarchical clustering analysis of EPZ-6438 deregulated genes highlighted the clustering of EZH2 inhibitor-treated PB with normal untreated PC (Supplementary Fig. S18). EPZ-6438 treatment appears to increase PC maturation with a PC gene expression signature in PB. Consistent with this result, a significant decrease of known B cell specific genes expression (including *MYC*, *CD58*, *CD22*, *AICDA*, *CD80*, *CXCR4*, *CD83*, *CIITA*, *CXCR5*, and *PAX5*) [1, 2, 4, 22] and an increase of known PC actors (such as *CD274*, *IL10*, *CCR2*, *CD38*, *TET1*, *FRZB*, *ID3*, *IRF1*, or *BMI1*) [1, 2, 4, 22] was observed (Supplementary Fig. S19). At a cellular level, the percentage of prePBs at Day 4 was not affected by the EPZ-6438 (Fig. 7a). Conversely, at Day 7, the percentage of prePBs was significantly reduced whereas PB percentage was significantly increased under EZH2 inhibition compared to control (Fig. 7a). Furthermore, PBs expressed higher levels of CD38 at their surface (Fig. 7b), suggesting a more advanced differentiation status. At Day 10, the percentage of PBs was significantly reduced, while the percentage of mature PCs increased after EPZ-6438 treatment (Fig. 7a) together with a higher CD38 and CD138 expression (Fig. 7b) characterizing a more mature state of EPZ-63438 treated PCs. Of interest, CD138/SDC1 is a PC gene associated with H3K27me3 and EZH2 suggesting a direct regulation of CD138/SDC1 expression by PRC2 during B to PCs differentiation (Supplementary Fig. S21). Moreover, analysis of immunoglobulin secretion showed a 3-fold increase in IgG secretion at Day 10 after EZH2 inhibition (Fig. 7c), while the PC production was only slightly increased (Supplementary Fig. S20). IgM production was significantly decreased after treatment in Day 10 PCs (Supplementary Fig. S20). We validated these results using two other PRC2 specific inhibitors: GSK-126, a potent, highly selective, S-adenosyl-methionine-competitive, small-molecule inhibitor of EZH2 methyltransferase activity [23] and MAK-683 inhibitor that binds to EED and disrupts the PRC2 complex [24]. These molecules are currently used in clinical trials [25]. Cell viability is not significantly affected after treatment with this two EZH2 inhibitors (Supplementary Fig. S24A), while, as expected, the levels of H3K27me3 are decreased (Supplementary Fig. S24B). The percentage of prePBs was significantly reduced whereas PB percentage was significantly increased under PRC2 inhibition during the first steps of B to PC differentiation (Supplementary Fig. S24C). A significant increase in the percentage of mature PCs after GSK-126 or MAK-683 treatment in association with a higher CD38 expression was also identified (Supplementary Fig. S24C&D).

**Fig. 8:**
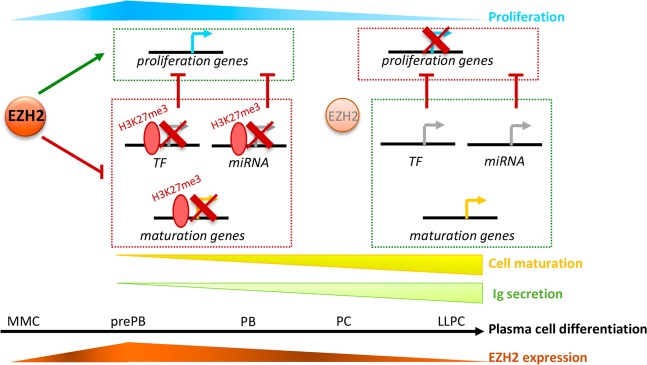
EZH2 controls prePB proliferation and maturation through gene regulation: Chart synthetizing EZH2 role in PCD. After MBC activation, *EZH2* expression is enhanced in prePBs. In this proliferative stage, EZH2 represses PC maturation genes through H3K27me3, and activates genes involved in proliferation, either directly or indirectly through H3K27me3-mediated inhibition of miRNA and transcription factors targeting cell cycle regulators. EZH2 expression is lost at the same rate as proliferation decreases and PC maturation occurs. We hypothesize that the absence of EZH2 allows the activation of PC maturation genes and the repression of genes involved in proliferation regulation

These data show that EZH2 inhibition or PRC2 targeting, through upregulation of PC genes and an earlier repression of B cell specific genes and genes involved in cell cycle, accelerates PC differentiation and Ig secretion (Fig. 8).

## Discussion

PC are rare cells with early differentiation stages taking place in anatomic locations that hamper full biological characterization, particularly in human. Herein, using an in vitro PCD model, we provide direct evidence that EZH2 is involved in late PC differentiation and biological functions. EZH2 is required for B cells to form germinal centers through repression of cyclin dependent kinase inhibitors and control of GC B cell proliferation [15]. Here, we demonstrated that EZH2 controls transcriptional changes during PC differentiation controlling B and PC genes through PRC2 and H3K27me3 dependent mechanisms. The significant induction of EZH2 in the transitional preplasmablastic stage was not documented before, to our knowledge. We previously described a regulatory network in which miR-106b inhibits the transcription repressor ZBTB4, known to target EZH2, that could explain, at least in part, *EZH2* upregulation in prePBs [10]. During lymphopoiesis, EZH2 is strongly expressed in proliferating cells, such as human germinal center B cells, cycling T and B lymphocytes and suggesting an important role in cell cycle regulation and in lymphocyte division [26]. In agreement with this hypothesis, lower levels of H3K27me3 and other histone methylation marks are observed in resting B-cells compared with activated and cycling B cells. In case of secondary immunization, MBCs can be restimulated to differentiate into highly proliferating preplasmalasts before differentiating into PBs and PCs [6]. Furthermore, several studies reported that EZH2 expression is linked to proliferation as a normal process [27]. The function of this association is to counteract the cell-division-mediated dilution of H3K27me3, to regulate the transcription of genes involved in cell cycle and to control DNA replication during S phase [28–30]. This could explain the high EZH2 expression identified in preplasmablasts.

EZH2 and H3K27me3 are associated with B and PC genes transcriptional repression in prePBs. 30.6% of B cells genes significantly downregulated in prePBs are associated with EZH2-mediated H3K27me3. Among them, key B cell genes were identified including *CIITA*, *BAMBI*, *BACH2*, *BCR*, *ID3*, or *SMAD3. CIITA* gene expression regulation mediated by EZH2 and H3K27me3 have been reported in Hela cells [31, 32]. Furthermore, PAX5-regulated genes including *ID3, BACH2, CD47, VAV3, CD40, IGF2, FLT3, CR2/CD21, CD72, EBF1, FCER2/CD23, LEF1*, and *CIITA* [33] are significantly downregulated in prePBs in association with EZH2-mediated H3K27me3. *PAX5* and *BCL6* gene expression decreases in prePBs with concomitant IRF4 and PRDM1 upregulation [6]. However, neither H3K27me3 nor EZH2 were recruited to *PAX5* and *BCL6* promoters. These results underline an indirect role of PRC2 and H3K27me3 in the downregulation of B cell transcriptional program in prePBs. EZH2-mediated H3K27me3 is also involved in concomitant PC transcriptional program repression. These results are consistent with recent data describing an increased accessibility of a set of primed promoters, associated with H3K27me3, of repressed genes in undivided murine naïve B cells [17]. Among H3K27m3-associated genes, we identified IRF4 target genes including *DUSP5, CAV1, NFIL3, UAP1, PAM, CFLAR, FKBP11, UBE2J1, TIMP2, GFPT1, SLAMF7, BMP6, AVPI1, CFLAR, and BSPRY* [21], but not IRF4 itself. Among them, *NFIL3, UBE2J1, FKBP11*, *ABPI1*, and *BSPRY* have been shown to participate in the ER stress response and UPR [34–40]. UAP1 and GFPT1 are involved in hexosamine pathway that take part in protein quality control mechanisms [41–44]. Therefore, PRC2, through H3K27me3, was found to negatively regulate genes that were involved in endoplasmic reticulum and Golgi apparatus functions known to play a key role in PC by accommodating the synthesis of secreted Ig. *XBP1* splicing is essential to support the unfold protein response gene program induction [45]. Our team previously highlighted that *XBP1* was mainly expressed as its unspliced isoform in prePBs [6]. This PRC2-mediated gene regulation concurs with the prePB status that starts to secrete Igs but at a lower level than PBs or PCs [6].

PrePBs are highly proliferating cells [6]. The repression of B and PC transcriptional programs is associated with the activation of proliferation. Our results underline for the first time a role of EZH2 in PC differentiation poising during the prePB stage through H3K27me3-mediated gene repression, concomitant with activation of proliferation. The proliferation signature is enriched in genes presenting EZH2-bound promoters without H3K27me3. A H3K27me3-independent EZH2-mediated transcriptional activation was previously reported in cancers [46, 47]. However, further investigations are needed to confirm a direct role of EZH2 in transcriptional gene activation during normal PCD. EZH2 overexpression inhibits DNA damage response pathways, allowing survival of activated GC B-cells during AID-mediated somatic hypermutation of Ig genes [16]. We reported a significant enrichment of genes involved in DNA repair associated with EZH2 and active transcription in prePBs that may play a key role to protect them from replicative stress. This data could be of interest to progress in the understanding of the poor outcome related to EZH2 overexpression in multiple myeloma [48–50].

The differentiation of B cells into PC takes place in a cell-division related manner [51] and is associated with DNA demethylation [52, 53]. Treatment with 5 azacytidine induces an increase of PC differentiation as reported here with EZH2 inhibitor [52, 53]. Our results agree with recent data demonstrating an enhanced antibody secreting cell formation for murine naïve B cells in presence of EZH2 chemical inhibition [17]. In this context EZH2 participates in the epigenetic mechanisms that could contribute to B to PC differentiation and cell fate selection [54].

Inhibition of EZH2 catalytic activity resulted in B to PC transcriptional changes associated with PC maturation induction. EZH2 inhibition reduced cell proliferation in prePBs and PBs, in addition to cell maturation and higher IgG secretion. However, the total number of PC obtained at day 10 is significantly lower in presence of EZH2 inhibitor. These data suggest that EZH2-mediated transcriptional regulation may be important to support and extend prePB amplification at the cost of PC differentiation. This is consistent with the rapid EZH2 downregulation observed in PBs and PCs together with proliferation inhibition and maturation. In vitro–generated PCs progressively died in our in vitro culture [6]. Human PC long-term survival requires addition of IL-6 in combination with at least APRIL and stromal cell-soluble factors, mimicking what is occurring in the putative PC niches [5]. Therefore, EPZ-6438 induces overexpression of genes related to apoptosis that are reversed by IL-6. The increased apoptosis identified at day 7 and day 10, after treatment by EPZ-6438, may be related to the stimulation of PC maturation reported in our study. The stimulation of PC differentiation mediated by EZH2 inhibition is associated with an earlier apoptosis of antibody secreting cells. According to that, EPZ-6438 treatment after differentiation of EZH2^high^ preplasmablasts into EZH2^low^ plasmablasts at day 7 did not affect final PC differentiation (Supplementary Fig. S22). We also validated our results using GSK-126, another potent, highly selective, S-adenosyl-methionine-competitive, small-molecule inhibitor of EZH2 methyltransferase activity [23] and MAK-683 inhibitor that bind to EED and disrupt the PRC2 complex [24] underlining that EZH2 inhibition or PRC2 complex disruption results in human plasma cell differentiation stimulation.

Our results support a model in which EZH2 is involved in the maintenance of prePBs/PBs transitory immature proliferative state through H3K27me3-dependent gene regulation. These data thus provide critical insights into epigenetic-mediated reprogramming events that sustain PC cell fate through cell division and proliferation.

## Supplementary information

Supplementary Figures

Supplementary Figure legends

Supplementary experimental procedures

Supplementary Table S1

Supplementary Table S2

Supplementary Table S3

Supplementary Table S4

Supplementary Table S5

Supplementary Table S6

Supplementary Table S7

Supplementary Table S8

Supplementary Table S9

Supplementary Table S10

Supplementary Table S11
