# Supplementary material for: EZH2 is overexpressed in transitional preplasmablasts and is involved in human plasma cell differentiation
Source: Leukemia. 2019 Feb 12;33(8):2047–60. doi: 10.1038/s41375-019-0392-1 (PMC6756037; doi:10.1038/s41375-019-0392-1)

Supplementary Figure S1: SUZ12 and EED are overexpressed in preplasmablasts

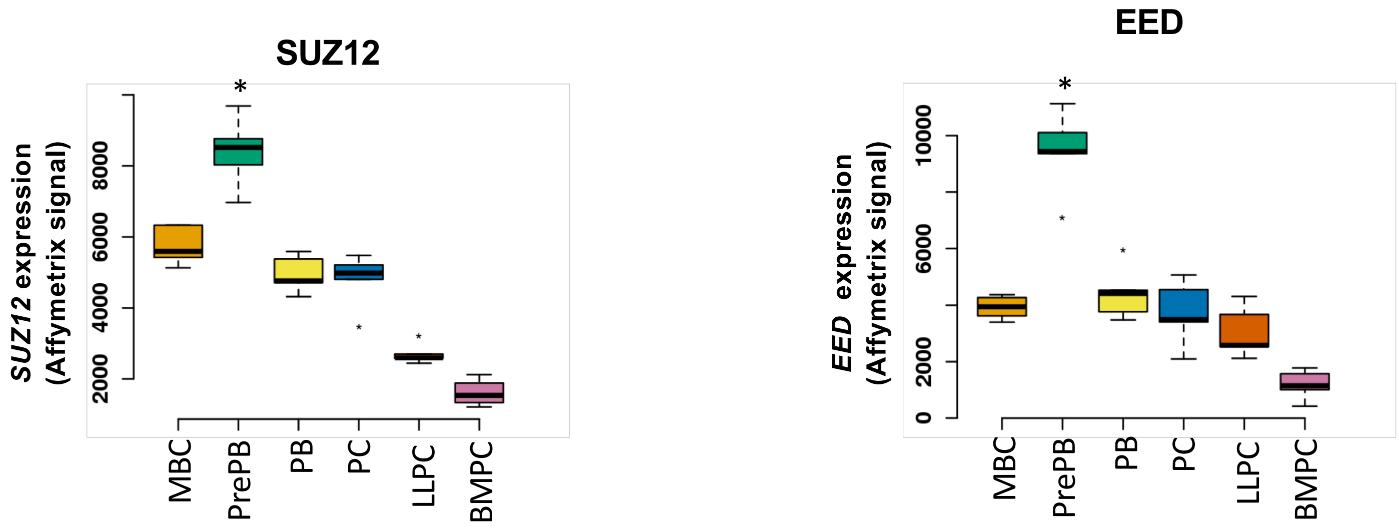

Supplementary Figure S2: Global H3K27me3 levels are stable during normal PCD

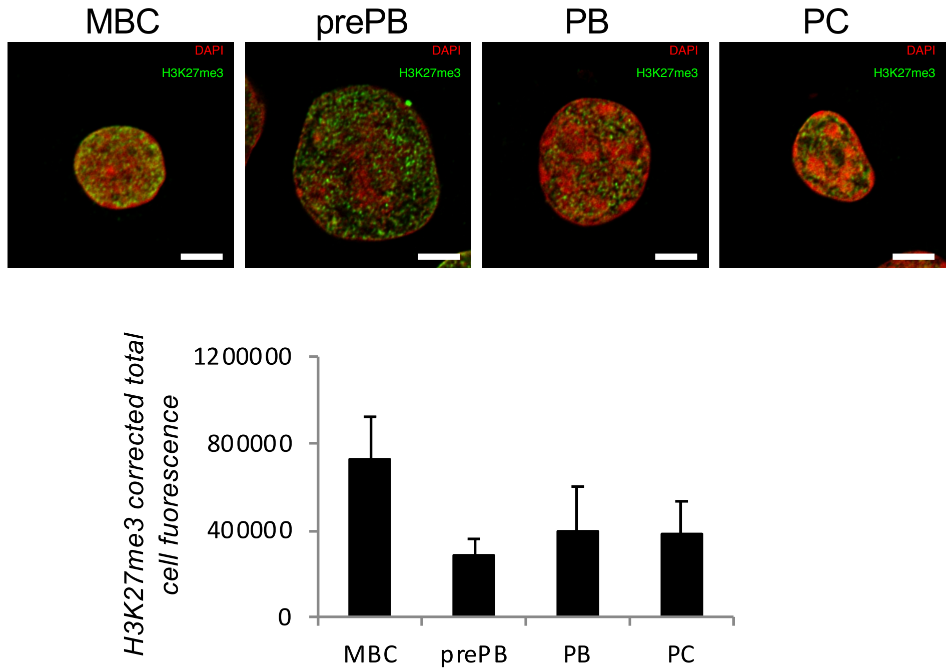

Supplementary Figure S3: EZH1 expression is anti-correlated with EZH2 expression from MBC to PC

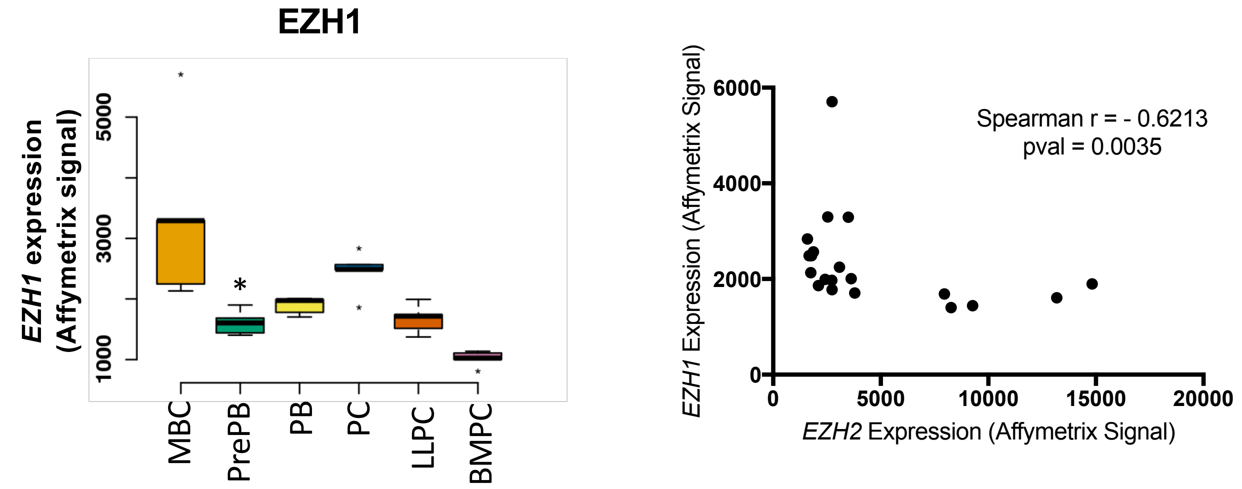

Supplementary Figure S4: EZH2 and H3K27me3 peak repartition across the genome in prePB and PB

	EZH2		H3K27me3	
	**prePB**	**PB**	**prePB**	**PB**
**Promoter (<=1kb)**	31.43%	26.7%	6.28%	7.79%
**Promoter (1-2kb)**	7.39%	5.47%	3.85%	4.44%
**Promoter (2-3kb)**	4.14%	3.02%	3.46%	3.99%
**5'UTR**	0.37%	0.26%	0.3%	0.3%
**3'UTR**	1.61%	1.39%	1.1%	1.12%
**1st Exon**	0.41%	0.44%	0.3%	0.33%
**Other Exon**	2.37%	2.29%	2.68%	2.41%
**1st Intron**	4.1%	4.25%	6.32%	6.1%
**Other Intron**	15.05%	17.79%	21.55%	20.25%
**Downstream**	1.23%	0.99%	0.98%	1.02%
**Distal Intergenic**	31.89%	37.38%	53.18%	52.25%

Supplementary Figure S5: EZH2 and H3K27me3 peak repartition around TSS in prePB and PB

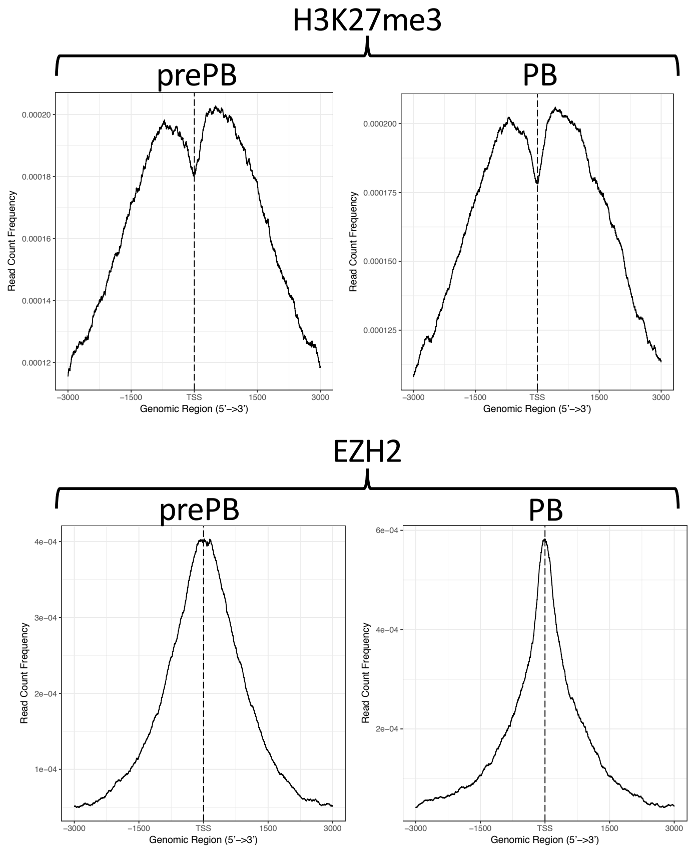

Supplementary Figure S6: Validated EZH2 target genes and pathways

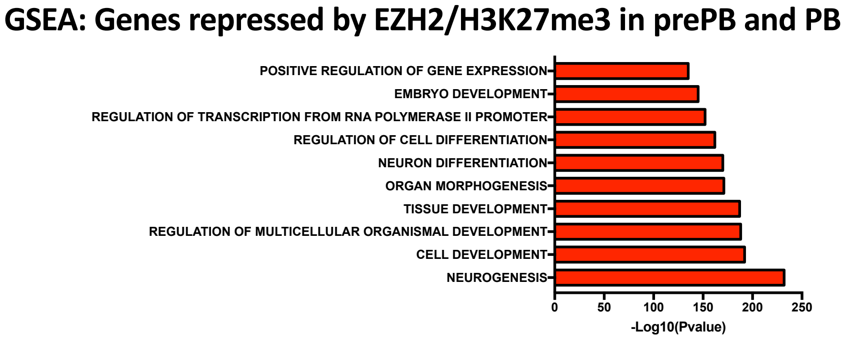

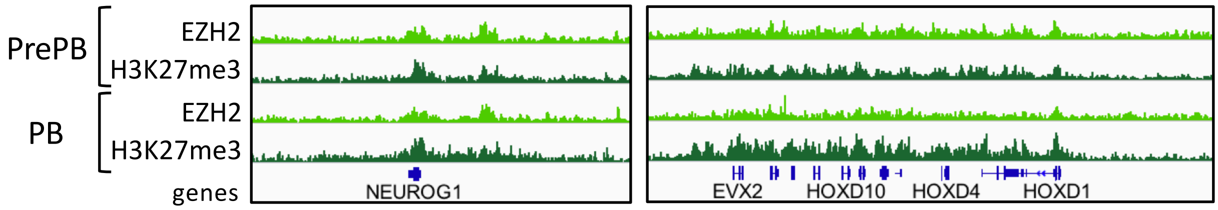

Supplementary Figure S7: Prediction analysis of transcription factor binding motif on EZH2o-bound genes in prePB and/or PB

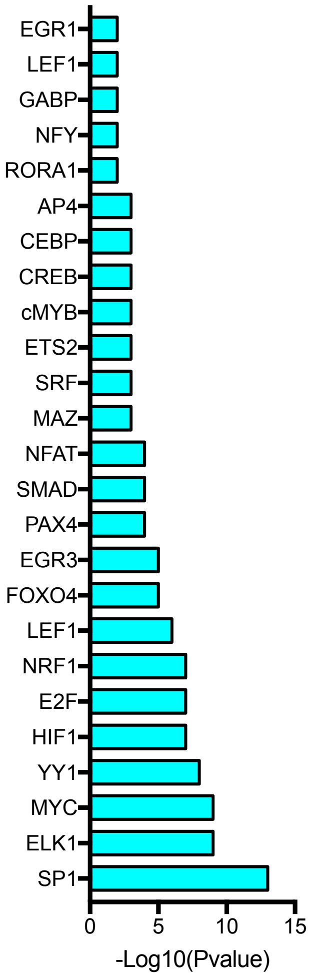

EZH2 vs.	Pearson r	P-value (two-tailed)	Significant? (alpha = 0.05)	PCD
CREB1	0,6687	<0,0001	Yes	MBC to BMPC
E2F1	0,7987	<0,0001	Yes	MBC to BMPC
ETS2	-0,3838	0,0363	Yes	MBC to BMPC
GABPB2	0,5575	0,0014	Yes	MBC to BMPC
HIF1A	-0,4821	0,0313	Yes	MBC to PC
MYB	0,9344	<0,0001	Yes	MBC to BMPC
NFATC3	0,4351	0,0163	Yes	MBC to BMPC
NRF1	0,5449	0,0019	Yes	MBC to BMPC
RORA	-0,3621	0,0492	Yes	MBC to BMPC
YY1	0,4073	0,0255	Yes	MBC to BMPC

Supplementary Figure S8: EZH2 regulation of B cell gene expression program in prePB and PB

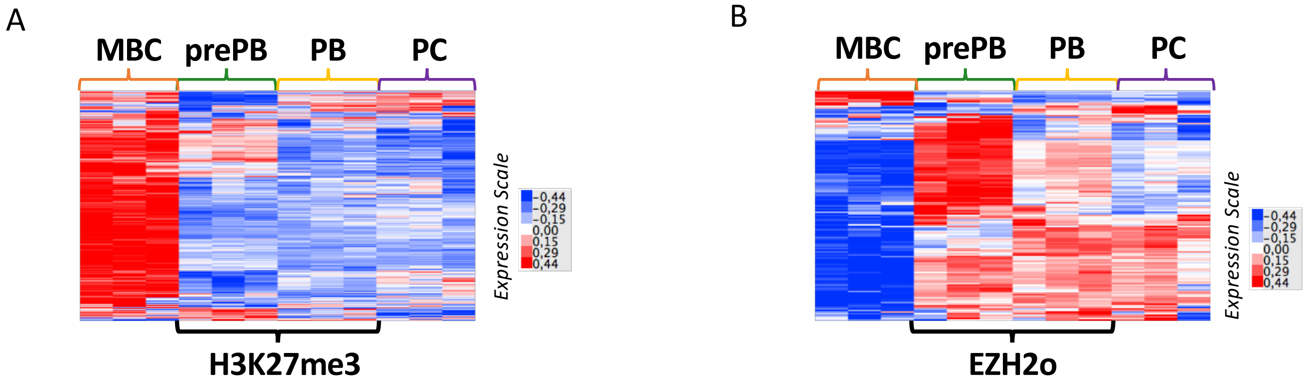

Supplementary Figure S9 : EZH2 regulation of plasma cell transcriptional program during PCD

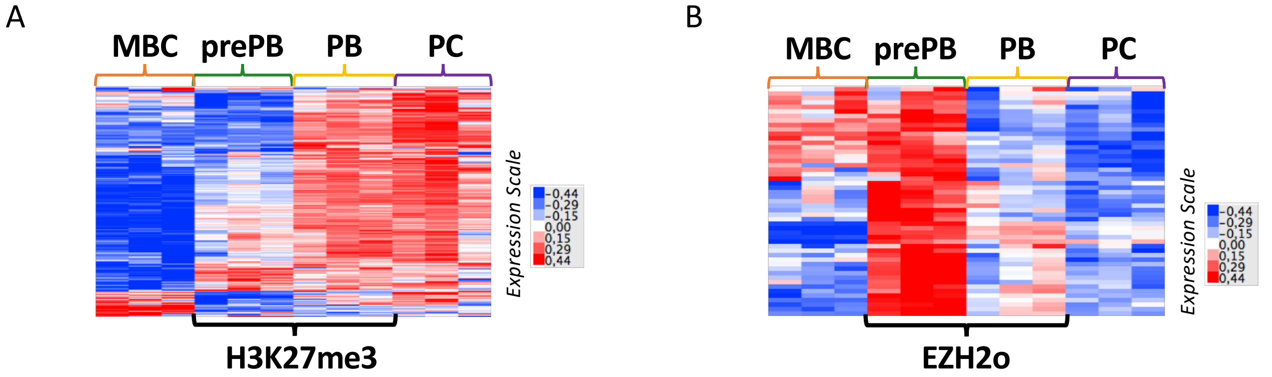

Supplementary Figure S10: EPZ-6438 treatment during PCD

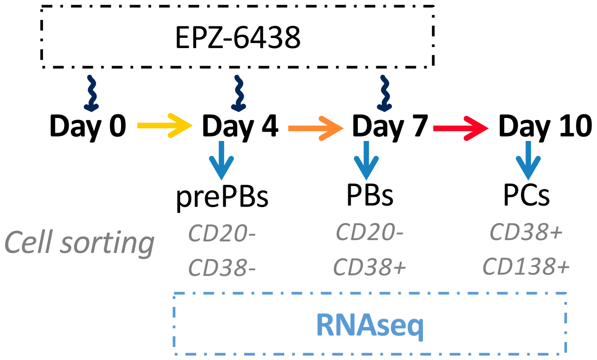

Supplementary Figure S11: EPZ-6438-induced H3K27me3 decrease

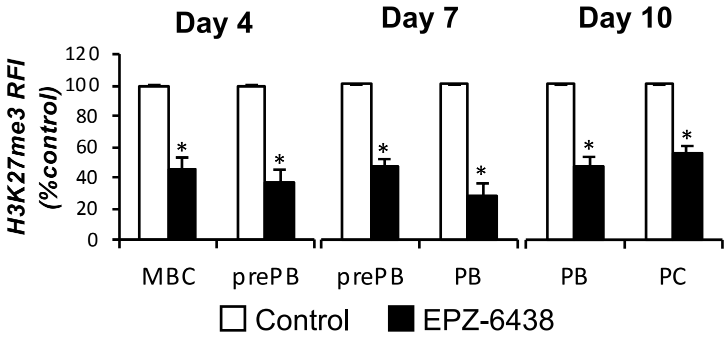

Supplementary Figure S12: EPZ-6438 decrease global H3K27me3 levels

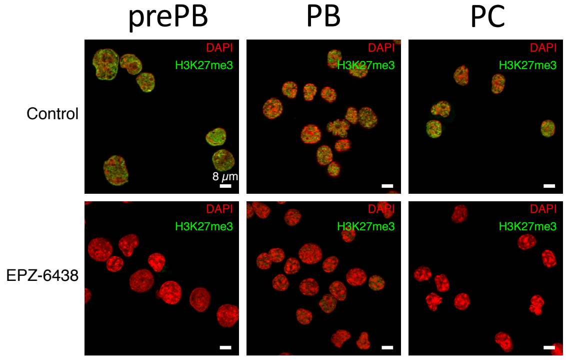

Supplementary Figure S13: EPZ-6438-upregulated genes during PCD

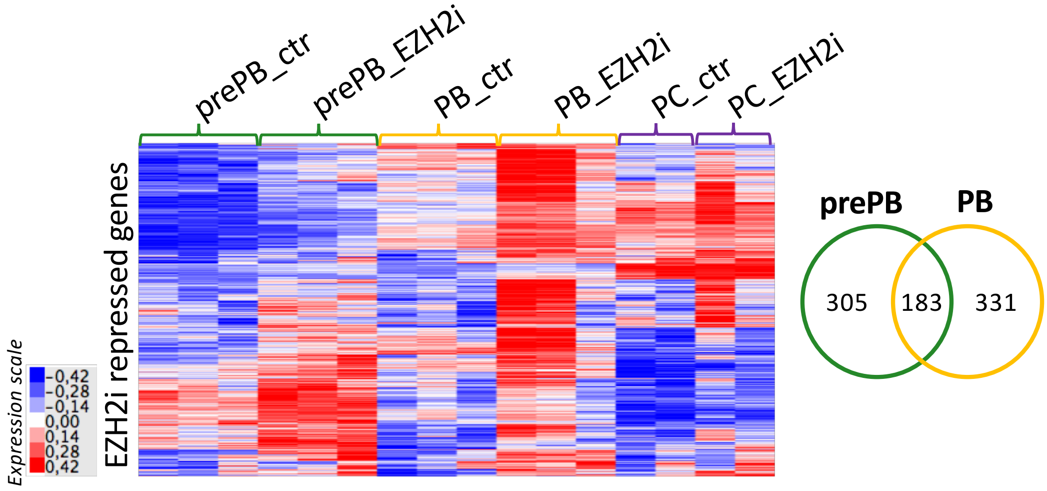

Supplementary Figure S14: EPZ-6438-downregulated genes during PCD

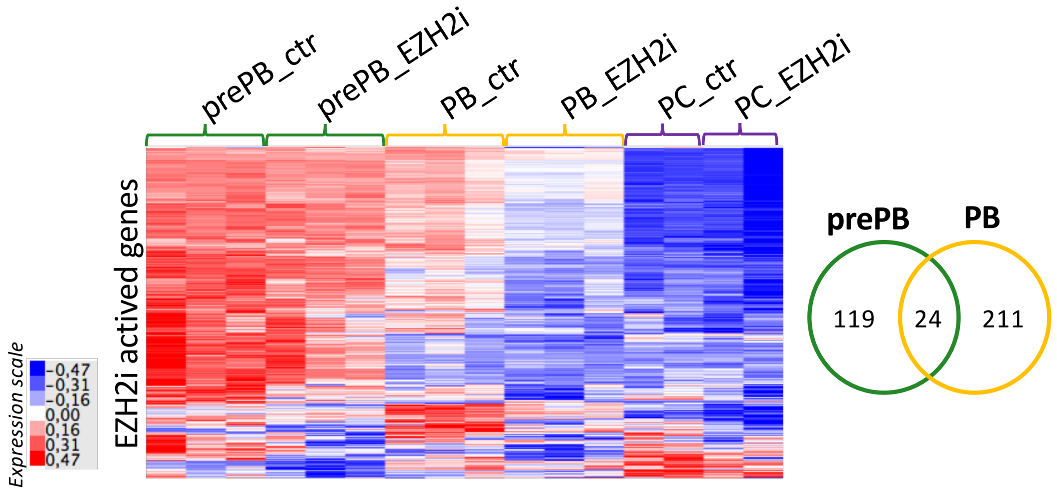

Supplementary Figure S15: EPZ-6438 deregulated genes in plasma cells

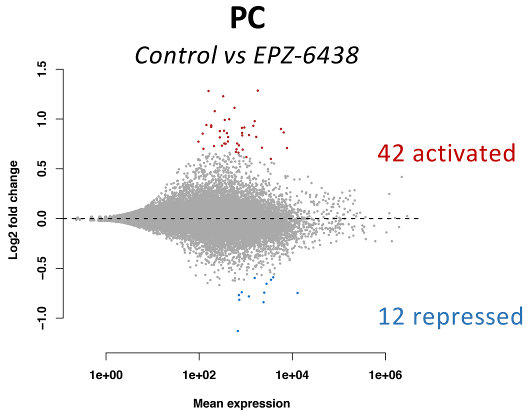

Supplementary Figure S16: Cell division under EPZ-6438

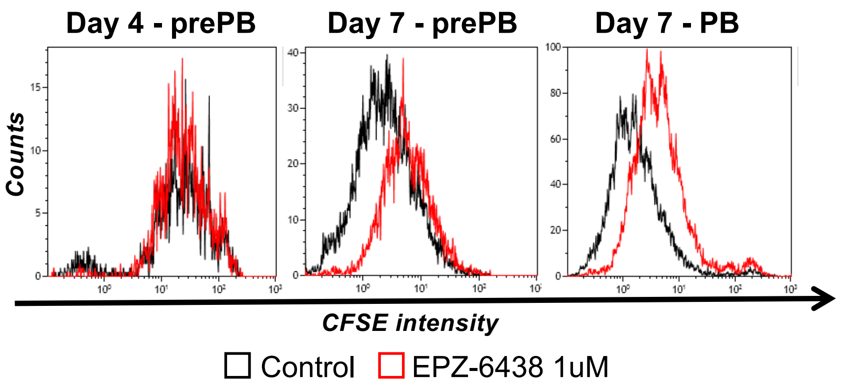

Supplementary Figure S17: EPZ-6438-induced DNA damage in PB and PC

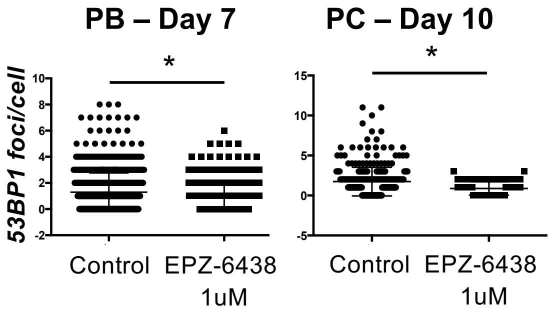

Supplementary Figure S18: Hierarchical clustering of EPZ-6438-deregulated genes during PCD

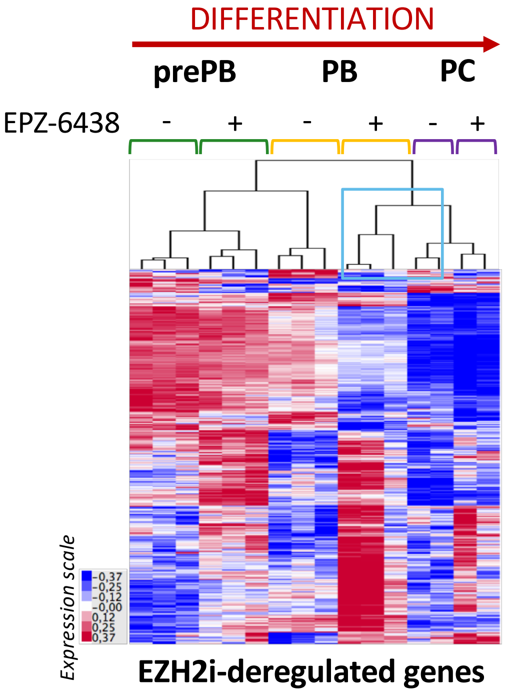

Supplementary Figure S19: PCD key genes deregulated by EPZ-6438 treatment

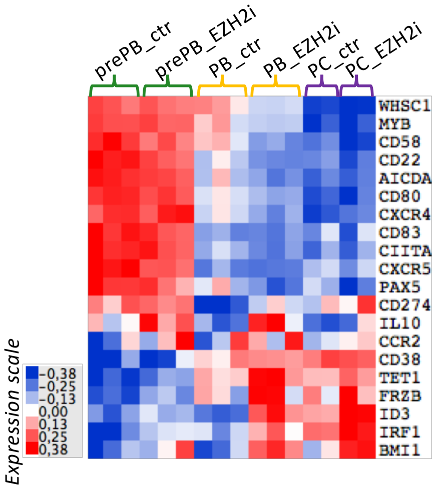

Supplementary Figure S20: Immunoglobulin production by PC after EZH2 inhibition

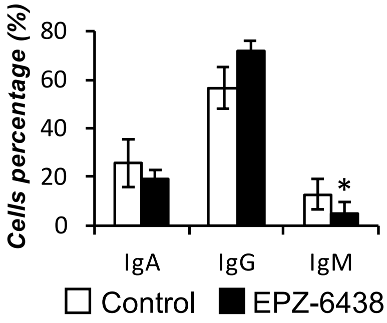

Supplementary Figure S21: EZH2 could directly regulate CD138 expression during B to PC differentiation

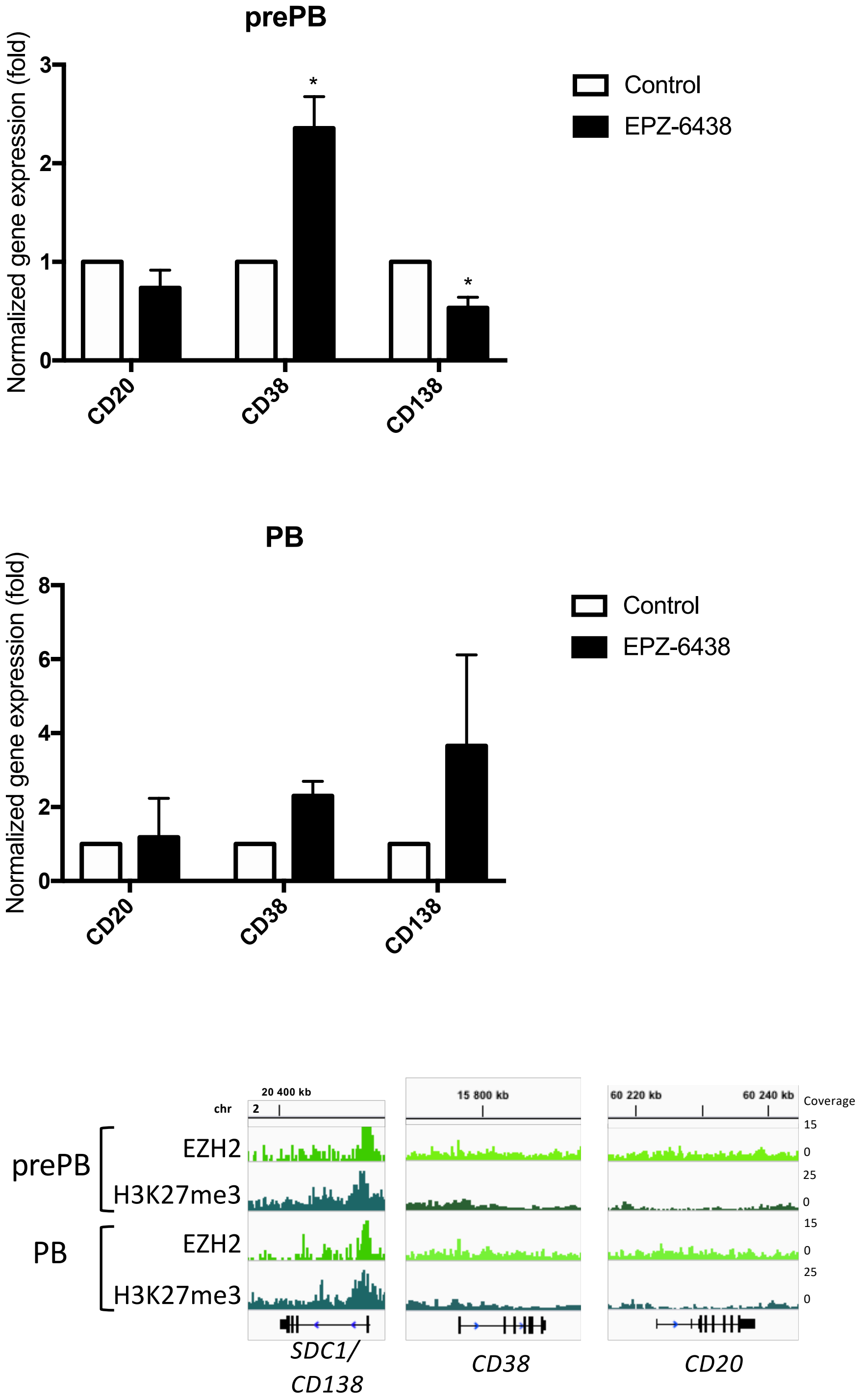

Supplementary Figure S22: EZH2 inhibition by EPZ-6438 at day 7 does not affect final PC differentiation

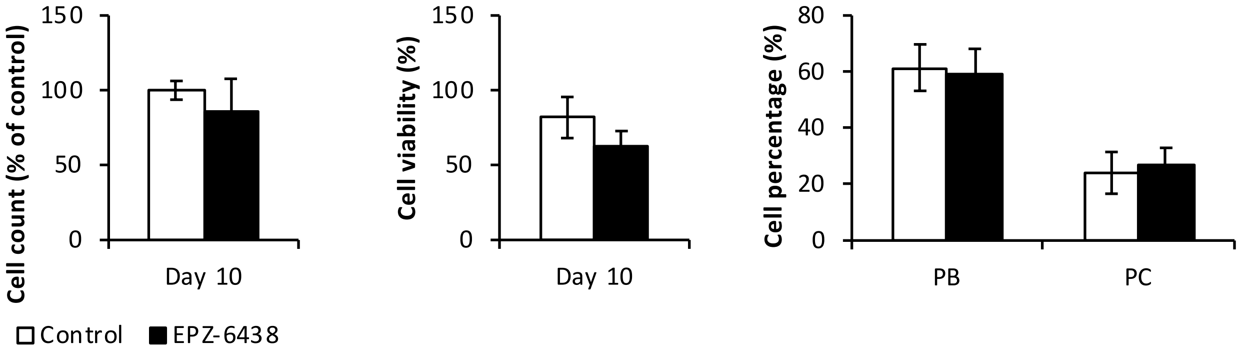

Supplementary Figure S23: EZH2 inhibition-induced cell death is partly Caspase-dependent at Day 10 of PCD

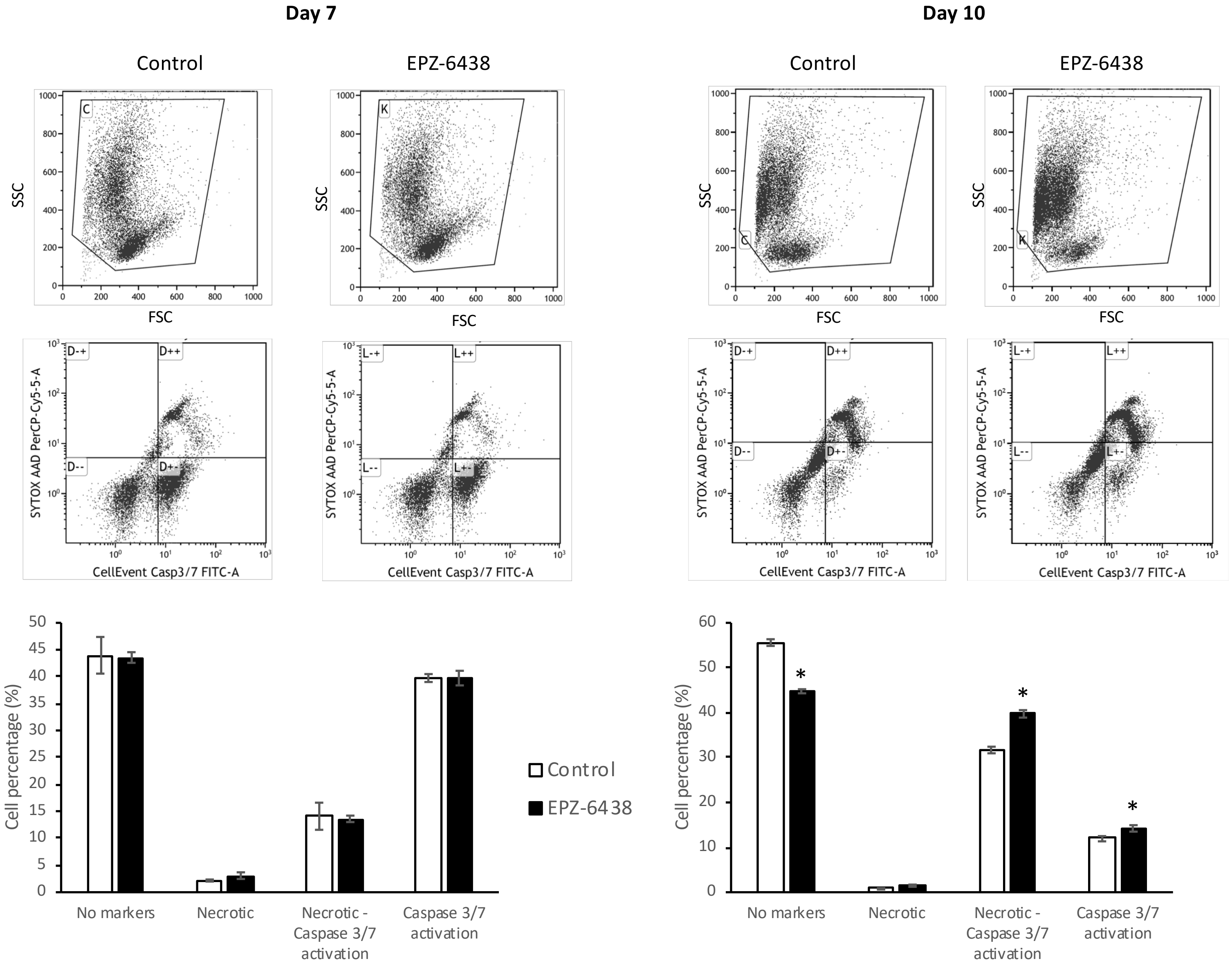

Supplementary Figure S24: PRC2 targeting accelerates PCD

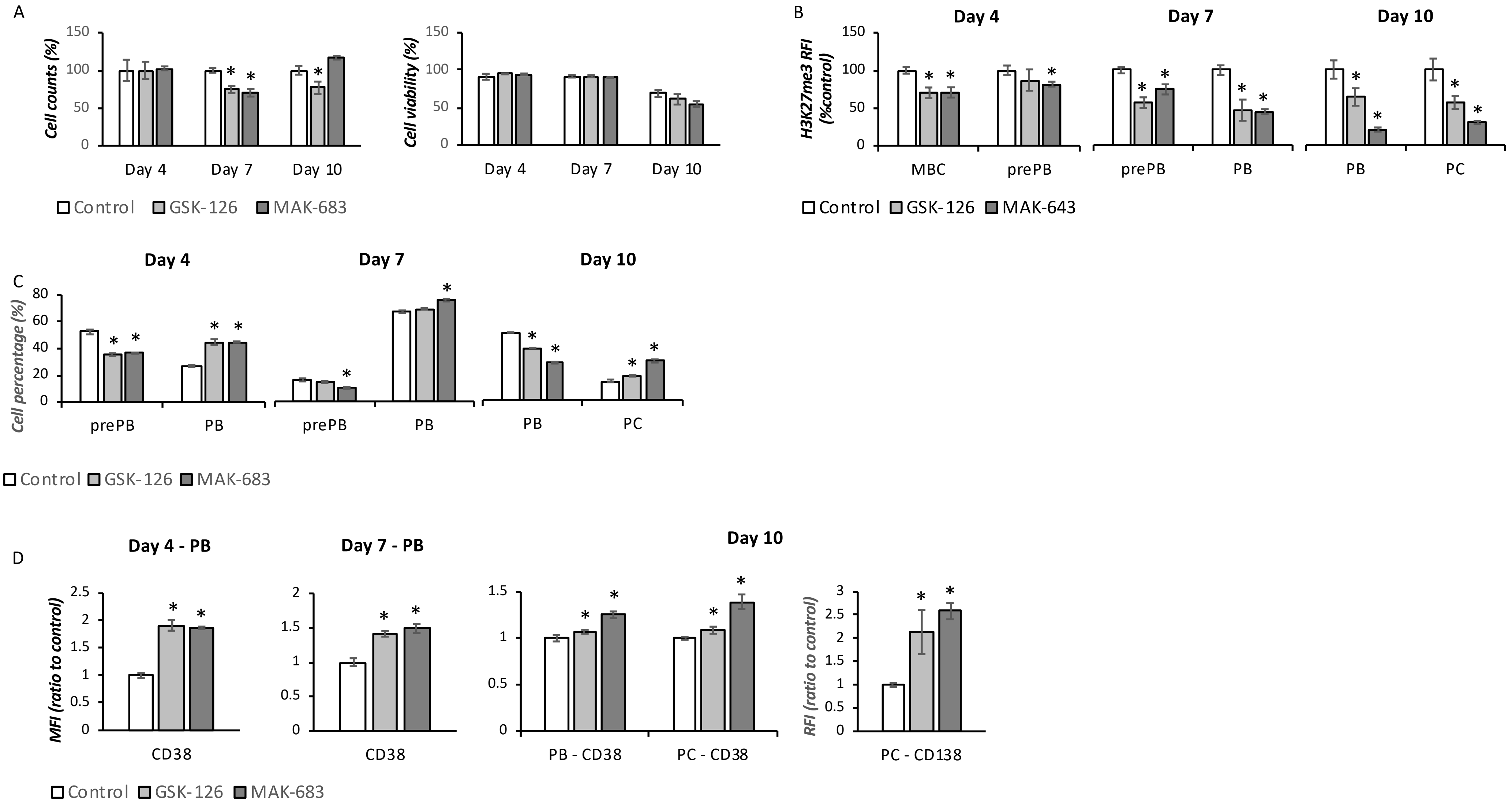

Supplement: Supplementary file 1 — Supplementary Figures [file 41375_2019_392_MOESM1_ESM.pdf]
