# Supplementary material for: EZH2 is overexpressed in transitional preplasmablasts and is involved in human plasma cell differentiation
Source: Leukemia. 2019 Feb 12;33(8):2047–60. doi: 10.1038/s41375-019-0392-1 (PMC6756037; doi:10.1038/s41375-019-0392-1)
Supplement: Supplementary file 2 — Supplementary Figure legends [file 41375_2019_392_MOESM2_ESM.docx]

**Supplementary figure legends**

**Supplementary Figure S1: *SUZ12* and *EED* are overexpressed in preplasmablasts:** *EED* and *SUZ12* Affymetrix microarrays expression signal during PCD.

**Supplementary Figure S2: Global H3K27me3 levels are stable during normal PCD:** H3K27me3 global levels were assessed by immunofluorescence in MBCs (Day 0), prePBs (Day 4), PBs (Day 7) and PCs (Day 10), using an anti-H3K27me3 antibody. Corrected total cell fluorescence (CTCF) was assessed using the ImageJ software (mean number of cells counted: 40).

**Supplementary Figure S3: *EZH1* expression is anti-correlated with *EZH2* expression from MBCs to PCs:** *EZH1* Affymetrix microarrays expression signal during PCD. Spearman correlation test of *EZH1* and *EZH2* expression in MBC, prePB, PB and PC shows a significant anti-correlation between the two methyltransferases (p-value = 0.0035).

**Supplementary Figure S4: Overall genomic distribution of EZH2 and H3K27me3 ChIP-seq peaks in prePBs and PBs:** This analysis was performed using MACS2 peak caller and ChIPseeker annotation tool. Table shows the percentage of H3K27me3 or EZH2 recruitment at different genomic regions.

**Supplementary Figure S5: EZH2 and H3K27me3 peak repartition around TSS in prePBs and PBs:** This analysis was performed using MACS2 peak caller and ChIPseeker annotation tool. Read count frequency was represented around genes TSS (-3000bp; +3000bp) for each ChIP experiments.

**Supplementary Figure S6: Validated EZH2 target genes and pathways:** GSEA pathway analysis of genes associated with H3K27me3 in prePBs and PBs. Log10(pvalue) was assessed for each pathway (FDR≤0.05). EZH2 and H3K27me3 peak distribution on *NEUROG1* gene and *HOXD* genomic locus in prePBs and PBs was visualized using IGV software.

**Supplementary Figure S7: Prediction analysis of transcription factor binding motif on EZH2o-bound genes in prePBs and/or PBs:** Transcription factors predicted to recognize and regulate genes associated with EZH2o in prePBs and/or PBs. Log10(pvalue) was assessed for each transcription factors. Pearson correlation test was performed on *EZH2* expression compared with *CREB1*, *E2F1*, *ETS2*, *GABPB2*, *HIF1A*, *MYB*, *NFATC3*, *NRF1*, *RORA* and *YY1* expression during PCD.

**Supplementary Figure S8: EZH2 regulation of B cell gene expression program in prePB and PB:** A) Heatmap representation of MBC upregulated genes expression during PCD. These genes are associated with the H3K27me3 repressive mark in prePBs and/or PBs. B) Heatmap representation of MBC downregulated genes expression during PCD. These genes are associated with EZH2o in prePB and/or PB.

**Supplementary Figure S9: EZH2 regulation of plasma cell transcriptional program during PCD:** A) Heatmap representation of PC upregulated genes expression during PCD. These genes are associated with the H3K27me3 repressive mark in prePBs and/or PBs. B) Heatmap representation of PC downregulated genes expression during PCD. These genes are associated with EZH2o in prePB and/or PB.

**Supplementary Figure S10: EPZ-6438 treatment during PCD:** Diagram describing the inhibition of EZH2 using the EPZ-6438 inhibitor in the previously described *in vitro* model of PCD. The effect of EPZ-6438 on the different populations –MBCs, prePBs, PBs and PCs – was assessed at the end of each differentiation steps (Day 4, Day 7 and Day 10). Each cell types were purified, according to previously described surface markers, to perform RNA sequencing.

**Supplementary Figure S11: EPZ-6438-induced H3K27me3 decrease**: H3K27me3 global levels after EPZ-6438 treatment (1uM) were analyzed by flow cytometry. Results are the mean value of the relative fluorescence intensity (RFI) ± SD of viable cells of 3 independent experiments. Statistical significance between conditions was assessed using Student paired t-test (*: pvalue<0.05).

**Supplementary Figure S12: EPZ-6438 treatment decreases global H3K27me3 levels:** H3K27me3 global levels were assessed by immunofluorescence in prePBs (Day 4), PBs (Day 7) and PCs (Day 10) after EPZ-6438 treatment, using an anti-H3K27me3 antibody.

**Supplementary Figure S13: EPZ-6438-upregulated genes during PCD:** Heatmap representation of EPZ-6438-activated genes expression during PCD. Expression scale shows highly expressed genes in red and poorly expressed genes in blue. Venn diagram stressing commonly and uniquely upregulated genes in prePBs (green circle) and PBs (yellow circle).

**Supplementary Figure S14: EPZ-6438-downregulated genes during PCD:** Heatmap representation of EPZ-6438-repressed genes expression during PCD. Expression scale shows highly expressed genes in red and poorly expressed genes in blue. Venn diagram stressing commonly and uniquely downregulated genes in prePBs (green circle) and PBs (yellow circle).

**Supplementary Figure S15: EPZ-6438 deregulated genes in PCs:** Scatterplot of EPZ-6438-deregulated genes in PCs. Activated genes are represented in red, while repressed genes are represented in blue.

**Supplementary Figure S16: Cell division under EPZ-6438:** Cells were labeled with CFSE at the beginning of the culture and the decrease in CFSE staining due to cell division was assessed at Day 4 and Day 7 for prePB and PB populations. Represented histograms are representative of 3 independent experiments. Statistical significance between conditions was assessed using Student paired t-test (*: pvalue<0.05).

**Supplementary Figure S17: EPZ-6438-induced DNA damage in PB and PC:** 53BP1 foci were observed in prePBs (Day 4), PBs (Day 7) and PCs (Day 10), with or without EPZ-6438 treatment. Number of foci per cell was quantified using ImageJ software (mean number of cells counted: 300). Presented charts are representative of 3 independent experiments. Statistical significance between conditions was assessed using Student paired t-test (*: pvalue<0.05).

**Supplementary Figure S18: Hierarchical clustering of EPZ-6438-deregulated genes during PCD:** EPZ-6438-deregulated genes based hierarchical clustering tree of treated and untreated prePBs, PBs and PCs.

**Supplementary Figure S19: PCD key genes deregulated by EPZ-6438 treatment:** Heatmap representation of the RNAseq expression of PCD key genes deregulated under EPZ-6438 treatment in prePBs, PBs and PCs. Expression scale shows highly expressed genes in red and poorly expressed genes in blue.

**Supplementary Figure S20: Immunoglobulin production by PC after EZH2 inhibition:** Percentage of CD138+ PC producing IgA, IgG and IgM in control and treated conditions, assessed by flow cytometry in 3 independent experiments.

**Supplementary Figure S21: EZH2 could directly regulate CD138 expression during B to PC differentiation:**  CD20, CD38 and CD138 gene expression PrePBs and PB in opresence or absence of EPZ-6438. Genomic snapshots of EZH2 and H3K27me3 ChIP-seq results on *CD20, CD38* and *CD138* genes in prePBs and PBs.

**Supplementary Figure S22: EZH2 inhibition by EPZ-6438 at day 7 does not affect final PC differentiation:** Day 7 cells were treated with 1µM EPZ-6438 and cell count, cell viability and PB/PC percentages were assessed at day 10 (3 independent experiments).

**Supplementary Figure S23: EZH2 inhibition-induced cell death is partly Caspase-dependent at Day 10 of PCD:** Cells were treated with EPZ-6438 throughout PCD, and caspase 3/7 activity was assessed by flow cytometry. Represented data are the mean percentage values ± SD of 3 (Day 7) and 3 (Day 10) separate experiments.

**Supplementary Figure S24: PRC2 targeting accelerates PCD**: A) After GSK-126 or MAK-683 treatments (2µM), cell counts and viability were analyzed by trypan blue assay. Represented data are the mean percentage of the absolute counts or viability ± SD of 3 independent experiments. B) H3K27me3 global levels were assessed by immunofluorescence in prePBs (Day 4), PBs (Day 7) and PCs (Day 10) GSK-126 or MAK-683 treatments (2µM), using an anti-H3K27me3 antibody. C) . Mean percentage ± SD (3 separated experiments) of MBC and prePB at Day 4, prePBs and PBs at Day 7; and PBs and PCs at Day 10 after GSK-126 or MAK-683 treatments (2µM). D) Protein expression of surface markers CD38 in PBs (Day 4, 7 and 10) and CD138 in PCs (Day 10) was assessed by flow cytometry with or without GSK-126 or MAK-683 treatments (2µM). Results are mean values of the relative fluorescence intensity (RFI) ± SD of viable cells of 3 independent experiments.
