# Supplementary material for: EZH2 is overexpressed in transitional preplasmablasts and is involved in human plasma cell differentiation
Source: Leukemia. 2019 Feb 12;33(8):2047–60. doi: 10.1038/s41375-019-0392-1 (PMC6756037; doi:10.1038/s41375-019-0392-1)
Supplement: Supplementary file 3 — Supplementary experimental procedures [file 41375_2019_392_MOESM3_ESM.docx]

**Supplemental experimental procedures**

**ChIP-seq and data analysis**

Cells were cross-linked in formaldehyde at a final concentration of 1% for 8 minutes. All experiments reagents were included in the AutoTrue MicroChIP kit (Diagenode, Liege, Belgium). Sonication was performed using a Bioruptor Plus sonication devise (Diagenode, Liege, Belgium) under the optimal conditions to shear cross-linked DNA to fragments if 100-300 base pairs in length. ChIP was conducted with the IPStar Compact Automated System (Diagenode) and the AutoTrue MicroChIP kit (Diagenode, C01010130) and Auto iDeal ChIP-seq kit for Transcription Factors (Diagenode, C01010172). ChIP were performed starting from 100.000 cells per IPs for H3K27me3 ChIP, and 4 million cells per IPs for EZH2 ChIP. H3K27me3 ChIP was performed in duplicates. Crossed-linked DNA was incubated 13h with the antibody (Table 1) and 3h with the beads. After 5min washes, eluates was recovered and reverse cross-linked for 4h at 65°C. Samples were treated for 1h with RNAse at 37°C, prior to DNA purification with the Auto IPure kit v2 (Diagenode, C03010010). Libraries were performed using NEBNext Ultra Library Prep Kit for Illumina (New England Biolabs). ChIP-seq were performed with Illumina NextSeq500 technology (Helixio, Clermont-Ferrand, France) using the following parameters: single-ended, 50bp, 40 million reads. Sequenced fragments were mapped to the reference human GRCh37 genome using Bowtie2 aligner(47). Peak calling was performed using MACS2 peak caller(48,49). INPUT samples were used as controls for peak detection, with a q-value of 0.05 for H3K27me3 ChIP, and a p-value of 0.01 for EZH2 ChIP. Sequence annotation was performed using the software R (version 3.2.3; available from https://www.r-project.org) and R package ChIPseeker developed by BioConductor project (available from <https://www.bioconductor.org/>)(50,51). The Integrative Genomic Viewer (IGV) was used to visualize EZH2 and H3K27me3 enrichment on specific genes.

**RNA sequencing and data analysis.**

Total RNA of MBCs (n = 3), prePBs (n = 3), PBs (n = 3), PCs (n = 3) was obtained using QIAGEN kit. RNA sequencing (RNA-seq) library preparation was done with 150ng of input RNA using the Illumina TruSeq Stranded mRNA Library Prep Kit. Paired-end RNA-seq were performed with illumina NextSeq sequencing instrument (Helixio, Clermont-Ferrand, France). RNA-seq read pairs were mapped to the reference human GRCh37 genome using the STAR aligner(52). All statistical analyses were performed with the statistics software R (version 3.2.3; available from https://www.r-project.org) and R packages developed by BioConductor project (available from <https://www.bioconductor.org/>)(50). The expression level of each gene was summarized and normalized using DESeq2 R/Bioconductor package(53). Differential expression analysis was performed using DESeq2 pipeline. P values were adjusted to control the global FDR across all comparisons with the default option of the DESeq2 package. Genes were considered differentially expressed if they had an adjusted p-value of 0.05 and a fold change of 1.5. Heat maps of gene expression were generated using the Cluster and TreeView. Pathway enrichment analyses were performed using online curated gene set, gene ontology (GO) and transcription factors binding site collections on the Gene Set Enrichment Analysis software (http://software.broadinstitute.org/gsea/msigdb/index.jsp)(54,55).

**Inferring putative miRNA targets using the miRTarget R package**

We used the previously described miRTarget R package to automatize and facilitate the procedure of inferring and validating miRNA–mRNA regulatory relationships(10). miRTarget includes a procedure to easily identify the predicted and validated mRNA targets given one or more miRNAs as input (https://github.com/kassambara/miRTarget). This is performed based on the union of two popular databases, miRTarBase (a database of experimentally validated miRNA targets, http://mirtarbase.mbc.nctu.edu.tw/, release 6.0)(56,57) and miRecords (http://c1.accurascience.com/miRecords/, version: 27 April 2013)(58). The miRecords database includes validated miRNA targets and also an integrated sequence based

miRNA target prediction resource from 11 popular miRNA target prediction programs (diana, microinspector, miranda, mirtarget2, mitarget, nbmirtar, pictar, pita, rna22, rnahybrid, targetscan). We retained only experimentally validated target genes (in either miRecords and/or miRTarbase databases). We then compared these validated targets to EPZ-6438-repressed genes.

**Analysis of Ig secretion**

Culture supernatant were harvested at day 10 of the PCD after EPZ-6438 treatment. IgA, IgG and IgM concentrations were assessed by ELISA using human IgA, IgG and IgM kits from Bethyl Laboratories (Montgomery, TX, USA), according to manufacturer’s recommendations.

**Immunofluorescence microscopy**

After deposition on slides using a Cytospin centrifuge, cells were fixed with 4% PFA, permeabilized with 0.5% Triton in PBS and saturated with 5% bovine milk in PBS. Cytospins were incubated 60min per primary and secondary antibodies diluted in 5% bovine milk in PBS. After 15min DAPI incubation, slides were mounted with Vectashield Mounting Medium (Cliniscience, Nanterre, France). Images and fluorescence were captured with a ZEISS Axio Imager Z2 microscope (X63 objective) and ZEISS LSM780 Confocal microscope and analyzed with Omero (omero.mri.cnrs.fr) server and ImageJ software. Corrected total fluorescence (CTCF) was calculated by subtracting the background fluorescence intensity to the mean fluorescence intensity of each nucleus. CTCF of 20 to 50 nuclei per condition was assessed.

**Flow cytometry analysis**

Cells were stained with a combination of 2 to 3 mAbs conjugated to different fluorochromes, as indicated. For intracellular staining, cells were fixated and permeabilized with Cytofix/Cytoperm kit (BD Biosciences) and incubated with antibodies recognizing IgM, IgA, IgG or H3K27me3 antigen. Flow cytometry analysis was performed with a FACSAria cytometer using FACSDiva 6.1 (Becton Dickinson, San Jose, CA, USA) and with a Cyan ADP cytometer driven by the Summit software (Beckman Coulter). The Kaluza software (Beckman Coulter) was used for data analysis. The fluorescence intensity of the cell populations was quantified using the staining index (SI) formula: [mean fluorescence intensity (MFI) obtained for a given mAb minus MFI obtained with a control mAb]/[2 times the standard deviation of the MFI obtained with the same control mAb].

**CFSE labelling**

Cell division was assessed by CFSE labeling as previously described(6). Purified MBCs were washed and re-suspended in PBS/0.1% BSA with 5 μM CFSE (Molecular Probes, Eugene, OR, USA), incubated at 37°C for 10 minutes and extensively washed before culture. At day 4 of culture, cells were washed and labeled with anti-PB-CD20 and anti-PerCP-Cy5.5-CD38 for flow cytometry analysis. At day 7 of culture, cells were washed and labeled with anti-PB-CD20, anti-PerCP-Cy5.5-CD38 and anti-APC-CD138. Cell divisions were quantified using the ModFit LT software (Verity Software House, Topsham, ME, USA).

**Caspase 3/7 activity assessment**

After EPZ-6438 treatment, caspase 3/7 activity was assessed by flow cytometry using CellEvent^TM^ Caspase-3/7 Green Flow Cytometry Assay Kit (ThermoFisher Scientific, Invitrogen, Catalog Number C10427) using manufacturer protocol.
