# Supplementary material for: EZH2 is overexpressed in transitional preplasmablasts and is involved in human plasma cell differentiation
Source: Leukemia. 2019 Feb 12;33(8):2047–60. doi: 10.1038/s41375-019-0392-1 (PMC6756037; doi:10.1038/s41375-019-0392-1)
Supplement: Supplementary file 4 — Supplementary Table S1 [file 41375_2019_392_MOESM4_ESM.pdf]

Supplementary Table 1: Antibodies used for each experiments

Cytometry

Monoclonal Antibody	Dilution	Company	Reference
CD19-APC (clone HIB19)	20	BD Biosciences, Le Pont de Claix, France	555415
CD27-PE (clone M-T271)	20	BD Biosciences, Le Pont de Claix, France	555441
CD20-PB (clone B9E9)	20	Beckman Coulter (Fullerton, CA, USA)	A74777
CD20-FITC (clone B9E9)	20	Beckman Coulter (Fullerton, CA, USA)	A07772
CD38-PE	20	Beckman Coulter (Fullerton, CA, USA)	A07779
CD38-PerCP-Cy5.5	20	Beckman Coulter (Fullerton, CA, USA)	B49199
CD138-PE (clone B-A38)	20	Beckman Coulter (Fullerton, CA, USA)	A40316
CD138-APC (clone B-A38)	20	Beckman Coulter (Fullerton, CA, USA)	A87787
H3K27me3-AF647	50	Cell Signalling	#12158
IgA-PE	200	Southern Biotechnology (Birmingham, AL, USA)	2052-09
IgM-FITC	500	BD Biosciences, Le Pont de Claix, France	555782
IgG-FITC	500	Southern Biotechnology (Birmingham, AL, USA)	2043-02

Fluorescence Microscopy

Target	Dilution	Host	Company	Reference
EZH2 (D2C9) XP	500	Rabbit	Cell Signaling (Danvers, MA, USA)	#5246
H3K27me3	500	Rabbit	Active Motif (La Hulpe, Belgium)	#39156
53BP1	300	Rabbit	Novus Biologicals (Littleton, CO, USA)	NB100304
gH2AX	200	Mouse	Merk Millipore (Billerica, MA, USA)	05-636
Alexa Fluor 555 donkey anti-rabbit IgG	500	Donkey	Life Technologies (Carlsbad, CA, USA)	A31572
Alexa Fluor 488 mouse anti-rabbit IgG	500	Goat	Life Technologies (Carlsbad, CA, USA)	A11008

ChIP-seq

Target	Dilution	Company	Reference
H3K27me3 Premium	200	Diagenode (Liege, Belgium)	C15410195
EZH2 (D2C9) XP	50	Cell Signaling (Danvers, MA, USA)	#5246
