# Supplementary material for: EZH2 is overexpressed in transitional preplasmablasts and is involved in human plasma cell differentiation
Source: Leukemia. 2019 Feb 12;33(8):2047–60. doi: 10.1038/s41375-019-0392-1 (PMC6756037; doi:10.1038/s41375-019-0392-1)
Supplement: Supplementary file 11 — Supplementary Table S8 [file 41375_2019_392_MOESM11_ESM.pdf]

<u>**Supplementary Table S8 : EPZ-6438-downregulated genes associated with EZH2o**</u>

EPZ-6438-repressed genes in prePB associated with EZH2o in prePB	EPZ-6438-repressed genes in PB associated with EZH2o in prePB	EPZ-6438-repressed genes in PB associated with EZH2o in PB
ABCA7	ACD	C12orf57
RELB	AQP3	C9orf40
ZNF354A	BRCA1	CD58
	C12orf57	CPT1A
	CD58	DNM2
	CHAF1A	EXO1
	DCK	KIAA0922
	DTL	LCK
	EOGT	LIG1
	IMPA2	LIMD2
	ITPK1	MCM5
	LIMD2	MTHFD1L
	MYB	NBEAL2
	OSBPL10	NOTCH1
	PCK2	PIK3CD
	RASGRP2	RAD51AP1
	S1PR1	RAD54L
	SH3BP5	RASGRP2
	SNX22	RRM1
	ZNF85	SH3BP5
		TMSB10
		TRAF4
		VIM
		WHSC1
		ZNF714
